# Conflict Resolution for Mesozoic Mammals: Reconciling Phylogenetic Incongruence Among Anatomical Regions

**DOI:** 10.3389/fgene.2020.00651

**Published:** 2020-07-08

**Authors:** Mélina A. Celik, Matthew J. Phillips

**Affiliations:** School of Biology and Environmental Science, Queensland University of Technology, Brisbane, QLD, Australia

**Keywords:** Mesozoic mammals, correlated homoplasy, incongruence, australosphenida, haramiyida, Multituberculata

## Abstract

The evolutionary history of Mesozoic mammaliaformes is well studied. Although the backbone of their phylogeny is well resolved, the placement of ecologically specialized groups has remained uncertain. Functional and developmental covariation has long been identified as an important source of phylogenetic error, yet combining incongruent morphological characters altogether is currently a common practice when reconstructing phylogenetic relationships. Ignoring incongruence may inflate the confidence in reconstructing relationships, particularly for the placement of highly derived and ecologically specialized taxa, such as among australosphenidans (particularly, crown monotremes), haramiyidans, and multituberculates. The alternative placement of these highly derived clades can alter the taxonomic constituency and temporal origin of the mammalian crown group. Based on prior hypotheses and correlated homoplasy analyses, we identified cheek teeth and shoulder girdle character complexes as having a high potential to introduce phylogenetic error. We showed that incongruence among mandibulodental, cranial, and postcranial anatomical partitions for the placement of the australosphenidans, haramiyids, and multituberculates could largely be explained by apparently non-phylogenetic covariance from cheek teeth and shoulder girdle characters. Excluding these character complexes brought agreement between anatomical regions and improved the confidence in tree topology. These results emphasize the importance of considering and ameliorating major sources of bias in morphological data, and we anticipate that these will be valuable for confidently integrating morphological and molecular data in phylogenetic and dating analyses.

## Introduction

The mammalian crown group includes monotremes and therians (marsupials and placentals) and all extinct descendants of their most recent common ancestor. Mammals share characteristics such as lactation from mammary glands, hair (at least ancestrally), enucleate red blood cells, a muscular diaphragm, and a jaw joint formed by the dentary (mandible) and the squamosal (cranium). Defining Mammalia and its membership among the modern fauna is trivial. However, extending this task to fossils has proven far more elusive. From mammaliaform origins in the Triassic to the Jurassic, early mammals underwent profound morphological changes. These included the evolution of several character complexes that were upheld as high-value markers for defining mammalian taxonomic exclusion or inclusion but that have since been revealed to be homoplastic. For example, tribosphenic molars, which combine piercing/slicing and grinding functions, and detachment of the middle ear ossicles from the dentary have both now been shown to have evolved independently several times (Allin, 1975; Chow and Rich, 1982; Wang et al., 2001; Martin and Luo, 2005; Luo et al., 2007; Luo, 2011; Ramírez-Chaves et al., 2016).

Over the past three decades, the task of inferring relationships among Mesozoic mammals has shifted emphasis from intuitive form/function assessments of character complexes (*e*.*g*., Kemp, 1983; Szalay, 1993) to analyses of morphological character matrices (*e*.*g*., Rowe, 1988; Luo et al., 2002; Rougier et al., 2007; Luo et al., 2015). The hope with these increasingly large taxon sets of ever more numerous and finely distinguished characters is that the stochastic error is reduced, and long branches are sufficiently broken up by taxon sampling to tease apart phylogenetic signal from homoplasy. In practice, these theoretical benefits are tempered by extensive character incompleteness, temporal gaps in the fossil record, and models that do not closely reflect morphological evolution, such as assuming constant rather than episodic evolutionary rates across lineages and character complexes. Nevertheless, phylogenetic inference has been enhanced by a recent explosion in sampling Mesozoic mammals (especially from China), including early members of many clades (Meng, 2014; Luo et al., 2017; Zhou et al., 2019).

### The Generalized Insectivore Backbone of Mammal Phylogeny

This study focuses on reconstructing the affinities of dentally specialized or otherwise ecologically highly derived mammals. This endeavor nevertheless requires a well-resolved and reliable backbone of more generalized mammals. Plesiomorphic mammalian ecospace was occupied by relatively small insectivore/carnivores (Jenkins and Parrington, 1976; Kermack and Kermack, 1984), which also provided the basic body plan for major new waves of mammalian diversifications throughout the Mesozoic, from the Triassic morganucodont-like predecessors to the Cretaceous ancestors of the modern clades of Marsupialia and Placentalia. The ancestors of these therian clades (and potentially Mammalia) have typically been considered to have been terrestrial-scansorial (*e*.*g*., Jenkins, 1970; Kermack and Kermack, 1984; Krebs, 1991; Szalay, 1994). However, the ecological diversity of dietarily plesiomorphic Mesozoic mammals has turned out to be far more disparate for their locomotor modes, including fossorial (Luo and Wible, 2005; Luo et al., 2015), semi-aquatic (Ji et al., 2006), arboreal, and even gliding mammaliaforms (Luo, 2007).

It is a general consensus that taxa with ecological similarities tend to be close and even cluster in their morphospace distribution, as demonstrated by recent empirical studies (Chen and Wilson, 2015; Grossnickle, 2017; Benevento et al., 2019). In theory, this should assist with discriminating synapomorphy from homoplasy in phylogenetic inference (Hendy and Penny, 1989). There is less obvious potential for large-scale and correlated mandibulodental convergence if sampling only generalized insectivores. However, there is potential for cranial and postcranial convergence among some taxa, for example, with independent ecospace shifts into fossorial, semi-aquatic, or highly arboreal niches. There is also potential for parallelism (as distinct from convergence) between generalized insectivore lineages following evolutionary trends that were initiated before they diverged. Examples of such evolutionary momentum among generalized insectivores have been suggested for trends toward tribospheny (Butler, 1990; Davis, 2011) and more parasagittal posture (Ji et al., 1999). Overall, however, relative morphological conservatism and stationarity (similar evolutionary processes operating across lineages) should enhance congruence among anatomical regions and resolution overall for the placements of well-sampled, ecologically generalized insectivores in comparison to more ecologically derived taxa, such as monotremes, haramiyidans, and multituberculates.

As it turns out, most inferences of Mesozoic mammal phylogeny broadly agree on the relationships among at least the well-sampled, small insectivorous/carnivorous mammal clades (Szalay et al., 1993; Luo et al., 2002; Rougier et al., 2011). To summarize, morganucodonts and docodonts, which both retained the primitive dentary-attached middle ear bones and sprawling posture, are placed outside of crown Mammalia. The potentially paraphyletic eutriconodonts retained plesiomorphic “in-line” molar cusps but have more derived crania and postcrania and are placed on the therian stem lineage between docodonts and spalacotherioids (which have “reverse triangle” molar cusps). Closer still to the divergence of marsupial and placental therians are successive ranks of extinct clades of the cladotherian group, such as *Henkelotherium*, *Vincelestes*, and *Peramus*, among which can be traced the early development of the posterior (talonid) component of tribosphenic molars (Butler, 1990) and partly to fully coiled cochlea with internal bony structure to support the hearing organ for improved high- and low-frequency hearing (Manoussaki et al., 2008; Luo et al., 2011; Manley, 2018).

### Dietarily Apomorphic Mammals

Ecological deviations from the shrew-like archetype among Mesozoic mammals evolved across several lineages. We will later consider cranial and postcranial characters, but here we pay particular attention to the impact of substantial dietary apomorphy on inferring Mesozoic mammal relationships. The reasons for this are twofold: (1) All three major clades at the crux of current debates surrounding the temporal origin and the taxonomic composition of the mammalian crown group are mired in arguments over molar cusp homology. A key argument is whether the multi-cuspate molars and omnivory–herbivory adapted craniomandibular geometries of haramiyidans and multituberculates evolved convergently (Luo et al., 2015, 2017) or are indicative of shared ancestry as “allotherians” (Bi et al., 2014; Meng et al., 2014). The near-tribosphenic teeth of the Cretaceous monotremes, *Steropodon* and *Teinolophos*, and their stem australosphenidan relatives are also thought to have evolved independently of therian tribosphenic teeth (Luo et al., 2001). Some authors (Archer et al., 1993) have even preferred to use alternative akid (blade) terminology rather than cusp terminology for fossil monotremes to side-step the uncertainty in interpreting cusps on the trigonid and the talonid of molars; and (2) there is a strong prior expectation for extensive, correlated mandibulodental convergence associated with dietary evolution based on comparative anatomy of modern mammals and developmental genetics (Kangas et al., 2004; Springer et al., 2013). Moreover, mandibulodental characters comprise 41% of the data matrix that we employ (based on Huttenlocker et al., 2018), and so correlated homoplasy among these characters could present a profound phylogenetic bias.

The oldest australosphenidans appear to have been relatively generalized insectivores, including the earliest monotremes (*e*.*g*., *Teinolophos*). However, many mandibulodental characters scored for australosphenidans (including upper dental characters) were unable to be scored for the plesiomorphic taxa but were scored only for the highly specialized platypuses and echidna. It is in this context of potential phylogenetic influence that we refer to australosphenidans as dietarily or dentally apomorphic, alongside multituberculates and haramiyidans. The phylogenetic placement of dietarily apomorphic taxa has a strong bearing on the age of Mammalia. At present, the oldest fossils that can reliably be placed within the mammalian crown and can thus be employed to calibrate the monotreme-therian divergence are Middle Jurassic (∼163 Ma) in age, such as the cladotherian *Amphitherium* (*e*.*g*., dos Reis et al., 2012), or perhaps slightly older, based on the australosphenidan *Asfaltomylos* (Rauhut et al., 2002). If, however, early haramiyidans such as *Haramiyavia* are closely related to multituberculates and are crown mammals (*e*.*g*., Bi et al., 2014; Krause et al., 2014), then Mammalia has a Triassic origin, pre-dating ∼201 Ma. We aim to quantify the distribution of correlated homoplasy across the skeleton that arises upon the inclusion of dietarily derived taxa in the mammal tree. This may further inform the validity of combining data from different regions or support the preferential use of certain anatomical regions.

### Australosphenida and the Monotremes

Living monotremes include the semi-aquatic platypus (*Ornithorhynchus anatinus*) and the semi-fossorial echidnas (Tachyglossidae), all of which present extremely specialized morphological features. The divergence of monotremes from therians has been placed as remotely as among primitive therapsids (*e*.*g*., Simpson, 1959; Macintyre, 1967) to as recently as grouping with marsupials (*e*.*g*., Gregory, 1947; Kühne, 1973, 1977). More recent developments substantially narrow this range. One of these, as we will shortly outline, is the discovery of stem monotreme fossils, and the other is relaxed clock molecular dating, which strongly favors monotremes diverging just 20-40 million years prior to the divergence of marsupials and placentals (*e*.*g*., Kullberg et al., 2008; Phillips et al., 2009; Meredith et al., 2011). These timing estimates are consistent with recent morphological phylogenies, in which monotremes almost always diverge from therians closely before or after eutriconodonts (*e*.*g*., Rougier et al., 1996; Meng et al., 2006; Rowe et al., 2008), sometimes with other dentally divergent taxa, such as multituberculates (*e*.*g*., Meng et al., 2003) or the pseudo-tribosphenic shuotheriids (*e*.*g*., Luo et al., 2007).

Stem monotremes include the Cretaceous *Steropodon* (Archer et al., 1985), *Ausktribosphenos*, and *Teinolophos* (Rich et al., 1997, 1999) and their Jurassic cousins, *Ambondro* from Madagascar (Flynn et al., 1999) and *Asfaltomylos* (Rauhut et al., 2002) and *Henosferus* (Rougier et al., 2007) from Patagonia. This discovery of a Gondwanan radiation of (near)tribosphenic mammals precipitated the classification of a new infraclass, Australosphenida (Luo et al., 2001, 2002; Kielan-Jaworowska et al., 2004). Although these taxa are known predominantly from incomplete dentaries and dentitions, these early australosphenidans, which were less ecologically and morphologically specialized than living monotremes, have already overturned interpretations of key character complexes that were central to earlier debates on the placement of monotremes. In particular, the absence of near-tribosphenic molars in living monotremes led to a notion that monotremes would not be close to the ancestry of therians (*e*.*g*., Rowe, 1988). Conversely, the detachment of the middle ear bones from the mandible could be interpreted as evidence for linking monotremes to therians if fossils had been excluded (*e*.*g*., Miao, 1993). After the discoveries of tribosphenid-like molars in toothed monotremes from the Cretaceous (Archer et al., 1985; Rich et al., 2016) and the finding that the middle ear was still attached to the mandible (Rich et al., 2016), the absence of these features in living monotremes is now shown to be the result of evolutionary convergence in the ear and reversal of tribosphenic teeth in living monotremes.

The near-complete humerus from a stem monotreme, *Kryoryctes cadburyi* (Pridmore et al., 2005), gives the first fossil insights on the evolution of the humero-ulnar articulation in australosphenidans (Phillips, 2002; Pridmore et al., 2005). Modern monotreme humeri have a bulbous ulnar condyle superficially similar to those of early mammaliaformes and multituberculates. In *Kryoryctes*, the humero-ulnar articulation is intermediate between these convex ulnar condyles and the pulley-like ulnar trochlea morphology of modern therians. The concavity of this trochlea, although wide and shallow, is limited to the dorsal/posterior aspect, as for putative stem therians such as eutriconodonts (*e*.*g*., *Jeholodens*, Ji et al., 1999). Thus, the monotreme ulnar condyle may not be homologous with those of early mammaliaformes but instead derived from an ulnar trochlea, perhaps similar to those of close relatives of therians. Thus, even the fragmentary remains from Mesozoic australosphenidans are confirming long-held beliefs (Gregory, 1947; Phillips et al., 2009) that much of modern monotreme morphology is highly derived in association with their ecology rather than indicative of phylogeny. A key question now is whether the recent consensus on australosphenidan affinities lying close to eutriconodonts is underpinned by phylogenetic signal agreement across anatomical regions or is an emergent statistical property of averaging over incongruent phylogenetic signals.

### Combined Evidence and Interrogation of Homoplasy

Homoplasy among mammals is widespread across all anatomical regions (Sánchez-Villagra and Williams, 1998; Ji et al., 1999; Sansom and Wills, 2017). The so-called total evidence approach, in which all relevant characters are combined, has often been recommended on an assumption that signals from shared ancestry would prevail over homoplasies to recover the true phylogeny. The expectation is that the phylogenetic estimates may be more accurate when all characters are combined, even in the face of significant incongruence between data partitions (Kluge, 1989; O’Leary et al., 2003; Fitzhugh, 2006; Asher, 2007; Mounce et al., 2016). A series of new discoveries of early mammal fossils preserved with basicrania (*e*.*g*., Rougier and Novacek, 1998) and postcrania (*e*.*g*., Ji et al., 1999) have made it feasible to combine anatomical regions for total evidence phylogenetic analyses of Mesozoic mammals (*e*.*g*., Luo et al., 2002), but whether different anatomical regions would yield heterogeneous signals (Naylor and Adams, 2001) has received little examination for Mesozoic mammals. For paleontological studies, a total evidence approach can also help to add the less complete taxa that are nonetheless important for tracing the evolution of particular characters and for other reasons, *e*.*g*., being the earliest-known member of a clade (Luo et al., 2002; Kielan-Jaworowska et al., 2004).

Arguments against total evidence, in favor of taxonomic congruence (*e*.*g*., Miyamoto and Fitch, 1995; Naylor and Adams, 2001), are concerned that combining data partitions to “democratize” evidence ignores the issue of incongruence. Such masking of interaction effects is anathema for most fields of science and statistical analysis of variance, wherein understanding incongruence has long been the preferred path to resolution (Brown, 1975). Even among advocates of total evidence, there is room for excluding some characters from phylogenetic inference. Kluge (1989), for example, wrote that “including all relevant evidence can be seen as a harmless activity, unless one is prepared to argue *a priori* that certain evidence will confound the analysis and must therefore be eliminated.” The strongest advocacy for exploring incongruence and excluding data partitions has come from molecular phylogenetics, for which it has often been a relatively trivial matter to move beyond identifying the presence of incongruence to isolating the sources of misleading phylogenetic signals and identifying the underlying processes. Examples include compositional biases in mitochondrial DNA transition substitutions, which erroneously group monotremes with marsupials (Phillips and Penny, 2003), and convergent selection pressures phylogenetically grouping echolocating bats and dolphins based on the hearing gene, *Prestin* (Liu et al., 2010).

Molecular incongruence (such as noted above) at phylogenetic levels beyond the reach of deep coalescence and introgressive hybridization tends to stand out against a background of congruence among multiple, unlinked genes or is revealed by improved substitution modeling. These luxuries may be less applicable to morphological phylogeny. However, some morphological character complexes may be expected to be less reliable. Molar cusp patterns, for instance, often possess a combination of rapid evolution (which erodes phylogenetic signal) and functional/developmental correlations that can overwhelm the remaining phylogenetic signal (Luo et al., 2001; Kangas et al., 2004; Ramírez-Chaves et al., 2016). However, non-phylogenetic sources of character covariation in vertebrate morphology are numerous and commonplace, including (but not limited to) allometry, pleiotropic–developmental, and functional correlations associated with ecological convergence or evolutionary trends (parallelism), and their respective influences vary dramatically across skeletal regions (Goswami et al., 2014; Cardini et al., 2015). Hence, complex distributions of phylogenetic incongruence may emerge across anatomical region partitions (Sánchez-Villagra and Williams, 1998; Ji et al., 1999), which is not conducive to teasing apart correlated homoplasy from phylogenetic signal.

Maximum likelihood and Bayesian inference methods that attempt to model the evolutionary process can provide an improved probabilistic framework for tracing character transformation (*e*.*g*., Lewis, 2001; Ronquist, 2004; Lartillot and Poujol, 2011). In particular, they can accommodate evolutionary rate variation among regional partitions and taxa, including some forms of heterotachy (Kolaczkowski and Thornton, 2004)—shifts in character-specific evolutionary rates over time. However, the current models are not robust to functionally and developmentally correlated parallelism and convergence of character complexes. Most models assume that substitutions are independent and identically distributed. There are exceptions, such as the doublet model, which allows for correlation between paired RNA stem sites (*e*.*g*., Tsagkogeorga et al., 2009; Phillips et al., 2010). However, evolutionary covariation among morphological characters associated with allometry, other developmental correlations, functional constraints, and selection cannot be so simply modeled.

By partitioning analyses between anatomical regions, we hope to better accommodate evolutionary rate variation across characters and lineages and isolate major sources of correlated homoplasy. Moreover, we develop a parsimony-based method to infer the relative magnitude of correlated homoplasy among anatomical partitions that is attributable to specified taxa. Thus, by interrogating incongruence, we hope to better understand its biological underpinning and more robustly inform the placement of ecologically derived mammals.

## Materials and Methods

### Data

Our morphological dataset is based on Huttenlocker et al. (2018). The matrix consists of 537 morphological characters for 103 extinct mammaliaformes. Five deeper-diverging cynodonts are used as outgroups to root the phylogeny. Within australosphenidans, coding changes were made on contentious mandibular characters related to the presence of a postdentary trough and Meckel’s groove, following Ramírez-Chaves et al. (2016). We also included the near-complete humerus from *Kryoryctes*, which provides the only stem monotreme postcranial information. Two characters (body size and mandibular depth) that are phylogenetically informative among monotremes were included (from Phillips et al., 2009). The coding changes and justifications are provided in the Supplementary Material.

Taxon sampling for our initial analyses is informed by our aim to explore how incongruence between mandibulodental, cranial, and postcranial partitions varies with and without the inclusion of dentally specialized multituberculates, haramiyidans, and australosphenidans (for which crown monotremes contribute many mandibulodental and almost all cranial and postcranial characters). This requires a well-resolved backbone phylogeny of generalized insectivore/carnivores that are well sampled for characters across each of the three regional partitions. A near-complete, dietarily plesiomorphic (NCDP) taxon set was identified, which includes 27 taxa that are inferred to have been predominantly insectivorous/carnivorous. Taxa were only included in the corresponding NCDP_537_ dataset if they were represented by >55% completeness for the 537 characters, including >35% completeness for each of the three regions or alternatively >65% completeness overall if >35% completeness across only two regions. Inclusion in the latter case also required local stability on the overall tree (>95% maximum parsimony and maximum likelihood bootstrap support) in agreement with general consensus among recent studies. This phylogenetic stability criterion allows such taxa to be phylogenetically constrained for analyses on the region for which they are poorly complete so as to not unduly influence other taxonomic placements or homoplasy metrics for these regions. Including such taxa breaks up long branches and informs character transformation when data are missing among their close relatives.

### Initial Exploratory Analyses

A maximum parsimony (MP) backbone phylogeny was initially reconstructed in PAUP 4.0b10 (Swofford, 2002) for the 27 NCDP taxa. We focus on dietary plesiomorphy (generalized insectivore/carnivores) because postcranial data are sparse for many of the more derived taxa to be included later, and therefore locomotory plesiomorphy cannot be consistently inferred. The NCDP sampling comprised *Thrinaxodon*, *Pachygenelus*, and *Sinoconodon* as outgroups and then morganucodonts, docodonts, spalacotherioid symmetrodonts, eutherians, and metatherians, along with two potentially paraphyletic groups, “eutriconodonts” and “eupantotheres.”

Initial MP bootstrap analyses were also run on Australosphenida and then for the multicuspate clades, Multituberculata and Haramiyida. In each case, *Thrinaxodon*, *Pachygenelus*, *Sinoconodon*, and morganucodonts were retained as outgroups. One of the aims of the initial MP bootstrap analyses was to compare the phylogenetic resolving power, with all characters unordered or with 72 of the multistate characters ordered (see the Supplementary Material). Character ordering can potentially enhance the phylogenetic signal by effectively increasing the steps associated with evolving more distinct character states, under certain assumptions (Slowinski, 1993). MP bootstrap support comparisons are shown in Supplementary Table S1 and focus on groupings that are resolved in at least one of the ordered/unordered treatments, with between 60 and 95% bootstrap probability (BP). Other groupings are not considered because there is little benefit from either treatment if both give near full support or both are unable to resolve relationships. Among these comparisons, greater mean phylogenetic resolution was recovered under the ordered character treatment for the NCDP taxon set and for the Australosphenida taxon set (with and without *Kryoryctes*). Only the Haramiyida and Multituberculata analysis resulted in effectively the same support under both treatments. As such, from here on, we focus on analyses in which those 72 characters are treated as ordered, unless otherwise stated.

The MP bootstrap analyses were run with heuristic searches using random sequence addition with TBR branch swapping for 20 replicates across 1,000 pseudo-replications (full bootstrapping). In addition to the MP analyses, the phylogenies were also inferred under maximum likelihood (ML) and Bayesian inference for the NCDP taxa, including all 537 characters (we refer to this matrix as NCDP_537_) and a 53-taxon dataset that augmented these generalized insectivore/carnivores with the australosphenidans, multituberculates, and haramiyidans (we refer to this matrix as 53_537_) to denote both the number of taxa and characters. The ML analyses were conducted in IQ-TREE v1.6.11 (Nguyen et al., 2015) using the Mk + Gamma models (Yang, 1994; Lewis, 2001). One shortcoming of IQ-TREE is that the ordered and the unordered characters are separately partitioned, which increases parameterization with all branch lengths independently estimated for partitions (-sp option). The -spp option reduces parameterization because branch lengths are instead only proportionally scaled across partitions. Unfortunately, -spp precludes the biological realism of lineage-specific variation in branch lengths across partitions, which we expect for taxa that are, for example, dentally derived but postcranially plesiomorphic. As such, our main concern is whether it is more appropriate to separately partition the ordered and the unordered characters (six partitions: two for each of the mandibulodental, cranial, and postcranial regions) or employ only the three regional partitions with all characters unordered. Based on the primary 53_537_ dataset, the corrected Akaike information criterion (AIC) and the Bayesian information criterion (BIC) scores favor the three-partition scheme (3sp: –ln*L* = 8,430.2836, corrected AIC = 18,126.6648, BIC = 18,683.5067) over the six-partition scheme (6sp: –ln*L* = 8,078.5163, corrected AIC = 654,605.0326, BIC = 19,702.3355). Thus, we focus on the “3sp” ML analyses, which treat all characters as unordered. Results with ordered and unordered characters partitioned, but with proportionally scaled partition branch lengths, are provided in the Supplementary Material and Supplementary Table S2. Ultrafast bootstrap approximation (1,000 replicates) was used for assessing ML clade support.

Bayesian inference allowed ordered and unordered characters to be modeled within the same partition and was conducted with MrBayes 3.2.6 (Huelsenbeck and Ronquist, 2001). The Mkv model (Lewis, 2001) was employed with gamma-distributed rates across sites (G) for each of the mandibulodental, cranial, and postcranial partitions. Two independent analyses were run with three Markov chain Monte Carlo chains for 5,000,000 generations. The trees were sampled every 5,000 generations, with the first 25% discarded as burn-in. Clade frequencies across the two independent runs reached convergence (average standard deviation of split frequencies < 0.01), and the estimated sample sizes for the tree prior, likelihood, and posterior estimates for tree lengths and rate alpha parameters were >200 (Tracer v1.7.1; Rambaut and Drummond, 2014).

In all our exploratory analyses, the controversial haramiyids (or multituberculates), *Vintana* and Hahnodontidae (including *Cifelliodon*) were highly unstable in the tree (as outlined in our results) and substantially affected support at other nodes. Except where otherwise stated, these taxa from the 53_537_ data were subsequently excluded, resulting in the 51_537_ dataset.

### Testing Phylogenetic Congruence Between Anatomical Regions

The phylogenetic incongruence between anatomical region partitions was initially tested within a maximum likelihood framework. The 537 morphological characters were partitioned into three anatomical regions to determine the phylogenetic signal across the data: mandibulodental (220 characters), cranial (183 characters), and postcranial (134 characters). We subsequently consider correlated homoplasy among more localized sub-regions; however, these broader regional partitions provide greater statistical power. Tree topologies were estimated for the overall dataset (with ML models partitioned) and for each of the three anatomical regions. Congruence between topologies was assessed using Kishino–Hasegawa (KH), and approximately unbiased (AU) tests were implemented in IQ-TREE. Initially, this incongruence testing was applied to the NCDP_537_ and 51_537_ datasets. Our focus is on phylogenetic signals for relationships among the major groups and, therefore, to reduce the influence of alternative placements within major clades and control for this across all comparisons, constraints were applied within each of Morganucodonta, Docodonta, Gobiconodonta, Spalacotheroidea, Metatheria, Eutheria, Australosphenida, Multituberculata, and Haramiyida for groupings that received ≥ 90% bootstrap support on the overall dataset, in agreement with the general consensus among recent studies. The bootstrap support criterion was relaxed for constraining several taxa within the dietarily apomorphic clades, for which very few sampled characters limited the statistical power. Constraint trees are provided in the Supplementary Material. KH and AU incongruence testing was further applied to the placement of Australosphenida specifically on all internal branches for major groups on the NCDP_537_ tree.

Bayesian inference incongruence testing was undertaken in MrBayes with the same constraints applied as for the ML analyses described above. In these analyses, the mandibulodental, cranial, and postcranial characters were again modeled separately under Mkv + G as partitions that included both ordered and unordered characters. For each of the NCDP_537_ and 51_537_ datasets, the three partitions were initially constrained to share the same topology. Analyses were then run with different topologies allowed for each anatomical region partition. The 95% higher posterior densities (HPDs) for marginal likelihood were compared between these topologically linked and unlinked analyses in Tracer.

### Homoplasy Within Anatomical Regions

**FIGURE 1:**
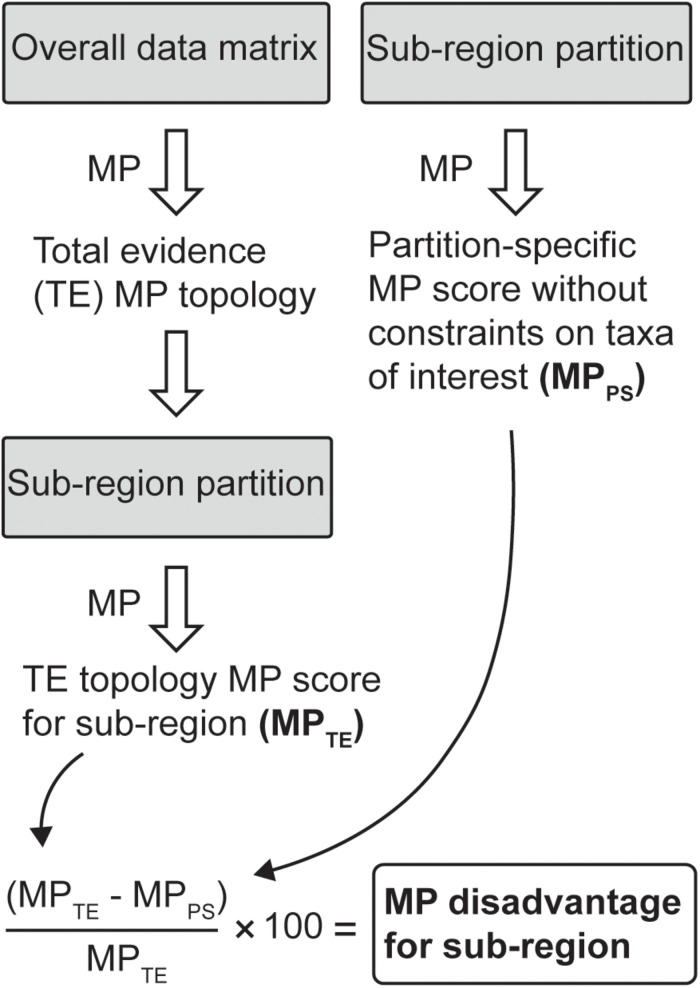
Workflow for calculating the maximum parsimony disadvantage for a data partition with the tree constrained to the total evidence topology, relative to unconstrained placements for taxa of interest (in this case, australosphenidans, multituberculates, and haramiyidans).

To more closely identify potential sources of correlated homoplasy induced by including the apomorphic clades, we present an MP-based metric, “MP disadvantage.” The method is set out in Figure 1. First, we partitioned the data into 10 finer sub-regions (with the number of characters indicated in parentheses): mandibular (34), cheek teeth (163), other dental characters (23), basicranial (117), calvariaviscerocranial (68), shoulder girdle (24), axial (16), pelvic girdle (13), forelimb (17), and hindlimb (62). For each sub-region, the taxa within the 53_537_ dataset that were scored for fewer than 10 characters were deleted. For the remaining taxa, the most parsimonious trees were inferred in PAUP on the full 51_537_ dataset, and the minimum number of tree steps for these overall favored (total evidence) topologies was then inferred on the relevant sub-region data only, giving the total evidence MP score (MP_TE_) for that sub-region. The minimum-length tree was then inferred for this taxon set on the sub-region data alone, without topological constraints on the taxa of interest and thus giving the partition-specific MP score (MP_PS_) for that sub-region. We refer to the percentage tree-length difference between these MP_TE_ and MP_PS_ tree scores as the MP disadvantage of a sub-region being constrained to the total evidence topology.

Maximum parsimony disadvantage is an indicator of the correlated homoplasy among sub-regions that is attributable to including the australosphenidans, multituberculates, and haramiyidans since the NCDP_537_ backbone constraint was employed for MP inference of the overall and sub-region trees. This constraint tree includes relationships among the 27 near-complete, dietarily plesiomorphic taxa that attained >90% MP bootstrap support (and are also generally agreed upon in recent analyses). Sub-regions with fewer than 20 characters (forelimb, axial, and pelvic girdle) were deemed to be unreliable and were not included in the main analysis. Fewer taxa could be included for these three sub-regions (*i*.*e*., with at least 10 characters sampled), and furthermore, these sub-regions provided high variation around the expected values (Supplementary Table S3). Since evolutionary modularity studies (*e*.*g*., Goswami et al., 2009) suggest that mammalian forelimbs, axial skeletons, and pelvic girdles are not functionally or developmentally closely correlated, it is also not appropriate for these sub-regions to be combined.

### Correlated Homoplasy Reduction and Extension to Less Complete Taxa

The cheek teeth and shoulder girdle sub-regions were identified as contributing disproportionately high levels of correlated homoplasy upon adding haramiyidans, australosphenidans, and multituberculates into the backbone phylogeny of generalized insectivore/carnivores. To assess the impact of these sub-regions on phylogeny, we re-ran the 51-taxon analyses with the cheek teeth (163 characters) and shoulder girdle (24 characters) excluded, leaving 350 characters. A more inclusive 78-taxon dataset was also compiled upon lowering the taxon completeness requirement to at least 15% of the 350 characters. This allowed the effect of excluding major apparent sources of correlated homoplasy to be evaluated for a broader phylogenetic context, including for the placement of several important but less well-known taxa close to the mammalian and therian crown nodes, such as the proposed sister of australosphenidans, the Shuotheriidae (Kielan-Jaworowska et al., 2002), and the putatively oldest eutherian, *Juramaia* (Luo et al., 2011). Phylogenetic inference of these new 51_350_ and 78_350_ datasets employed MP, ML, and Bayesian inference, as described above.

## Results

### Exploratory Phylogenetic Analyses

**FIGURE 2:**
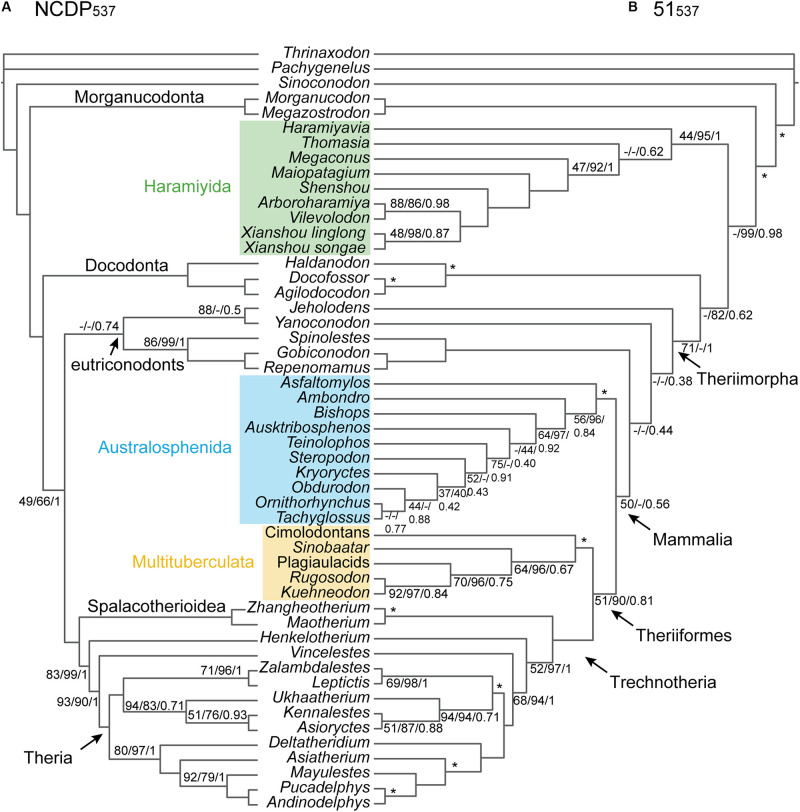
**(A)** NCDP_537_ and **(B)** 51_537_ phylogenies. The Bayesian inference topologies are shown, but with support values provided respectively for maximum parsimony-ordered (bootstrap), maximum likelihood (bootstrap), and Bayesian interference (Bayesian posterior probability) only for nodes at which at least one of these measures is < 95%. The asterisks indicate the constrained nodes (see Supplementary Material for the constraint used and Supplementary Figure S1 for the results from the unconstrained analyses). The dashes represent the branches which are not supported.

Initial phylogenetic analyses on the 27 highly complete “dietarily plesiomorphic” (NCDP_537_) dataset reconstructed a well-supported mammalian backbone phylogeny of generalized insectivore/carnivores (Figure 2A). The modern consensus is retrieved with Eutheria, Metatheria, Cladotheria, Trechnotheria, and Theriimorpha all strongly supported, and moving stemwise, more inclusive clades that successively include docodonts, morganucodonts, and then *Sinoconodon* all received maximum bootstrap support (BP_MP_ and BP_ML_) and Bayesian posterior probability (BPP). There is uncertainty regarding the relationships and potential paraphyly of the eutriconodonts; however, they are globally stable in that, among these generalized insectivore/carnivore taxa, eutriconodonts are all placed on the trechnothere stem lineage with maximum BP/BPP.

Separate MP bootstrap analyses were conducted for each of the main “dietarily apomorphic” taxa, Australosphenida, Multituberculata, and Haramiyida, rooted with stem-mammal outgroups. Strong support was recovered for key groupings within each of these clades (see the Supplementary Material). Within Australosphenida, the monotreme clade was recovered with 91% BP and, in turn, formed a clade (78% BP) with the other Australian australosphenidans, *Ausktribosphenos*, and *Bishops*. *Kryoryctes* was recovered among monotremes in all analyses with moderate support (>73% BP) despite only being included for 10 characters and not yet including several apomorphies with living monotremes that were noted by Pridmore et al. (2005) (see the Supplementary Material). Hence, we are confident of the placement of *Kryoryctes* with monotremes.

To enhance phylogenetic comparability and incongruence testing between mandibulodental, cranial, and postcranial regions, we focused on taxa that are well sampled across each of these regions. However, we also included several taxa that contribute much-needed information on otherwise less well-sampled sub-regions and that are locally stable on the overall dataset. These taxa can be reliably constrained in regional analyses so as to not unduly influence incongruence testing. The global stability of *Kryoryctes* and several mandibulodental taxa within Australosphenida (*Asfaltomylos*, *Ambondro*, *Ausktribosphenos*, *Bishops*, *Teinolophos*, and *Steropodon*) permits their inclusion for further analyses. Similarly, *Kuehneodon*, which is one of the oldest and most mandibulodentally complete multituberculates, did not meet the initial inclusion criteria but was stably placed as sister to *Rugosodon* (>90% BP) and therefore included in phylogenetic and incongruence testing analyses.

The controversial haramiyids or multituberculates, *Vintana* (Krause et al., 2014) and Hahnodontidae [including *Cifelliodon* (Huttenlocker et al., 2018)], were highly unstable in the tree. Although these two enigmatic taxa could contribute importantly to cranial character sampling, they are otherwise poorly known. MP and ML analyses respectively placed this Hahnodontidae–*Vintana* grouping outside Mammalia [nested within Haramiyida as sister to Eleutherodontidae, s*ensu* Huttenlocker et al. (2018)] and as sister to multituberculates, nested well within Mammalia. In contrast, our Bayesian inference analysis recovered weakly supported placements of *Vintana* with multituberculates and, separately, Hahnodontidae within Haramiyida. These two widely separated local optima also prevented MrBayes runs from converging on a global optimum. Improved sampling of non-cranial material from these two taxa or further cranial material from haramiyidans may resolve these affinities. However, our primary aim is to demonstrate and identify incongruence between data partitions. To isolate the impact of incongruence on phylogenetic estimates and to reduce the uncertainty and possible errors associated with *Vintana* and Hahnodontidae, these taxa were excluded.

With *Vintana* and Hahnodontidae excluded, phylogenetic analyses of the resulting 51_537_ dataset recovers Multituberculata and Haramiyida as reciprocally monophyletic [as in Huttenlocker et al. (2018)], with 93–100% support in ML and Bayesian inference, but with only 44–50% MP bootstrap support (Figure 2B). An important difference here may be that the ML and the Bayesian analyses partition the data by anatomical regions and allow evolutionary rates to vary across characters, thus effectively conferring greater weight to the influence of slower evolving characters. These more conserved characters typically retain more phylogenetic signal relative to non-phylogenetic signals, at least at deeper divergences (Philippe et al., 2000).

Including the dietarily apomorphic taxa with the highly complete plesiomorphic taxa substantially eroded the previously strong support for branching orders along the backbone of the tree, from crown Theria stemwards (Figures 2A,B). The resulting 51_537_ tree contains what is essentially a six-taxon polytomy that includes Multituberculata, Trechnotheria, Australosphenida, and three eutriconodont lineages, *Yanoconodon*, *Jeholodens*, and gobiconodontids. However, Theriiformes (Multituberculata and Trechnotheria) was recovered at >80% support by ML and Bayesian analyses of the 51_537_ data (Figure 2B and Supplementary Figure S1). Paraphyly of eutriconodonts was favored, with Gobiconodontidae, *Yanoconodon*, and *Jeholodens* falling successively deeper. Australosphenida was recovered as sister to Theriiformes (Multituberculata and Trechnotheria), albeit with weak support (34–50% BP_MP_, 32% BP_ML_, and 0.56–0.77 BPP). One further instability in the tree concerns the sister relationship to mammals (including eutriconodonts), which alternated between Docodonta or Haramiyida.

### Testing Phylogenetic Congruence Between Anatomical Regions

**FIGURE 3:**
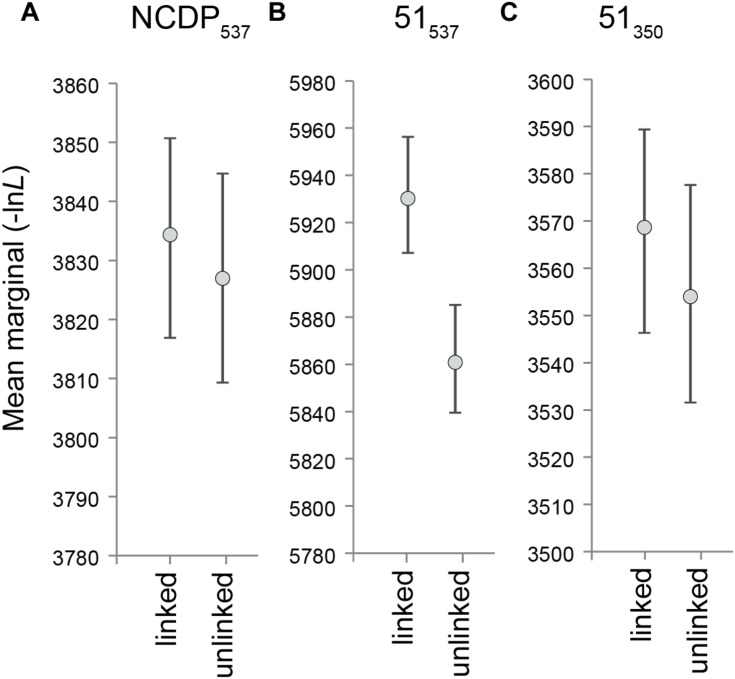
Bayesian inference marginal likelihood means and 95% higher posterior densities (averaged over two runs) for **(A)** NCDP_537_, **(B)** 51_537_, and **(C)** 51_350_, with the tree topology linked and unlinked across the three anatomical regions. ln*L* advantage = (M_Llinked_ – M_Lunlinked_)/M_Lunlinked_.

We investigated whether phylogenetic uncertainty in the affinities of the apomorphic australosphenidans, haramiyidans, and multituberculates relative to the 27 near-complete dietarily plesiomorphic taxa is substantially contributed to by phylogenetic incongruence between the mandibulodental, cranial, and postcranial anatomical regions. We found only a minor evidence of incongruence among the major NCDP_537_ clades. KH testing indicated that the topology of the overall (combined data) tree was not rejected in ML analyses of either the mandibulodental or cranial datasets but was rejected with the postcranial data (*P* = 0.0383, Table 1). Even in this case, the topological difference between the overall and the postcranial NCDP_537_ ML trees was minor, with the placement of *Yanoconodon* among eutriconodonts differing by a single branch step. Moreover, the analysis of NCDP_537_ in MrBayes provides widely overlapping likelihood 95% HPDs for treatments regardless of whether the topologies are linked or allowed to differ between the three anatomical region partitions (Figure 3A). This indicates close congruence between trees inferred from the different regional partitions for the NCDP_537_ data.

**TABLE 1:** Kishino–Hasegawa tests in IQ-TREE on the individual partition datasets (mandibulodental, cranial, and postcranial), assessing the congruence between the topology favored for each partition and the total evidence topology for NCDP_537_, 51_537_, or 51_350_.

	NCDP_537_		51_537_		51_350_	
Data used	*P*-value	Δln*L*	*P*-value	Δln*L*	*P*-value	Δln*L*
Mandibulodental	0.312	6.544	**0.0005**	82.768	0.0913	10.638
Cranial	0.138	6.1598	**0.0301**	21.766	0.0696	15.311
Postcranial	**0.0383**	7.3127	0.498	0.186	0.202	4.2128

*Bold values indicate the rejection (*P* < 0.05) of the NCDP_537_, 51_537_, or 51_350_ topologies on the mandibulodental, cranial, or postcranial characters.*

The inclusion of australosphenidans, haramiyidans, and multituberculates with the near-complete dietarily plesiomorphic taxa (51_537_) dramatically increased the incongruence among partitions. This is particularly salient for the Bayesian inference analyses in which the regional partitions are linked *versus* unlinked, with the 95% HPDs for the likelihoods becoming widely separated (Figure 3B). In KH testing, the overall (combined data) 51_537_ ML topology was rejected on both the mandibulodental (*P* = 0.0005) and the cranial data (*P* = 0.0301). The overall 51_537_ ML tree is not rejected on the postcranial data (*P* = 0.4980). However, the postcranial 51_537_ ML tree is rejected with both the mandibulodental and the cranial datasets (*P* < 0.0001). The strong phylogenetic incongruence between the anatomical regions is predominantly attributable to their alternative placements of the apomorphic australosphenidans, haramiyidans, and multituberculates. This is indicated by KH testing, providing almost identical results even without the backbone constraint being applied (see Supplementary Table S4).

**FIGURE 4:**
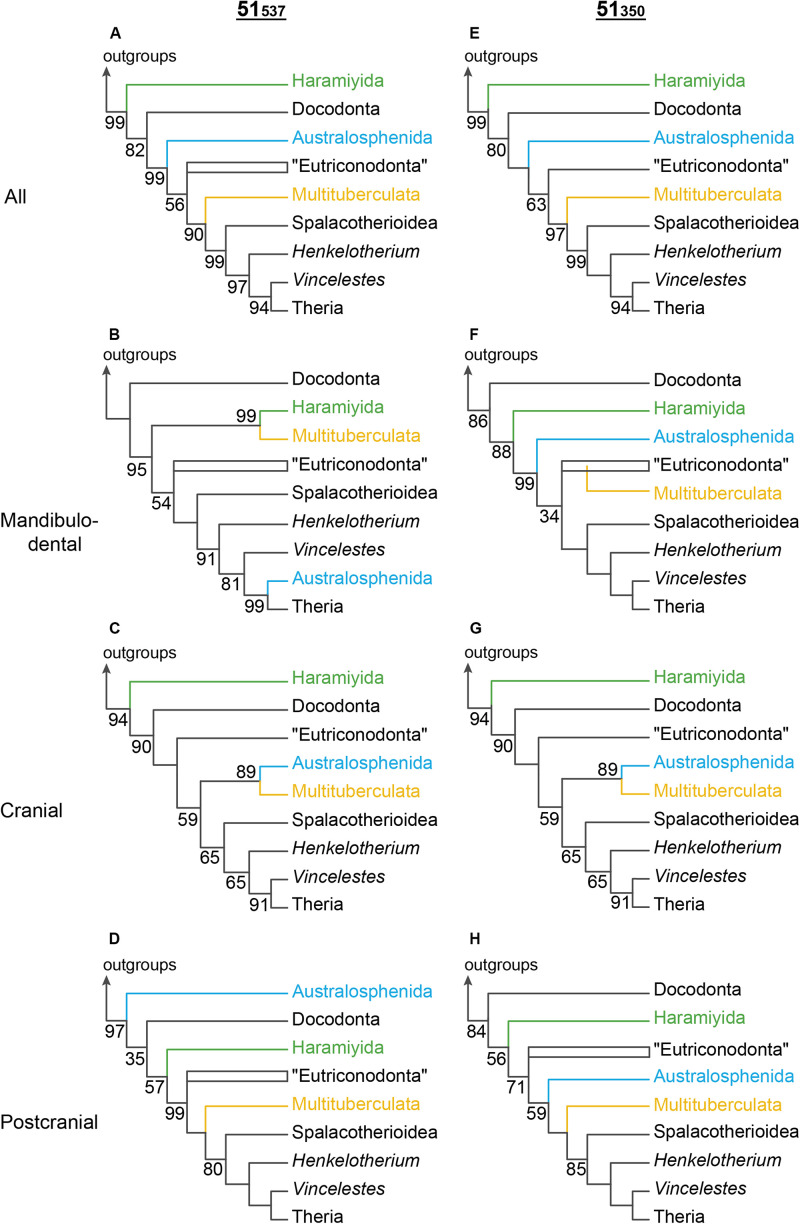
Alternative phylogenetic positions for Haramiyida (green), Australosphenida (blue), and Multituberculata (orange) for the 51_537_ and the 51_350_ datasets on **(A,E)** all characters combined, and separately **(B,F)** mandibulodental, **(C,G)** cranial, and **(D,H)** postcranial. These groups were constrained to be monophyletic, given that some taxa that are informative for the placement of the group have too few characters for one or more anatomical regions. The maximum likelihood bootstrap (BP_ML_) is represented only at nodes < 100%. The cranial trees are identical, since the same data is employed for this partition for both 51_537_ and 51_350_.

The topological manifestations of incongruence between the overall 51_537_ ML tree and regional trees for the placements of the dietarily apomorphic taxa on the NCDP backbone are shown in Figure 4. On the overall 51_537_ ML tree (Figure 4A), Haramiyida is strongly excluded from crown Mammalia (>99% BP). Similar haramiyidan placements (adjacent to Docodonta) are favored for the cranial region and for the postcranial region (albeit with australosphenidans deeper in the postcranial tree; Figure 4D). However, for the mandibulodental region, haramiyidans and multituberculates group together (99% BP, Figure 4B). Indeed when reciprocal monophyly was not enforced for these two multicuspate orders, haramiyidans were paraphyletic on the mandibulodental tree, with multituberculates sister to eleutherodontid haramiyidans.

Two australosphenidan placements are similarly likely on the overall 51_537_ dataset, as sister to Theriimorpha, or one step closer to therians, as sister to Theriiformes. However, the ML trees in Figure 4 show that the individual regions favor widely separated placements for Australosphenida. On the mandibulodental data (Figure 4B), australosphenidans are sister to crown Theria (99% BP), and on the cranial data (Figure 4C) they group with multituberculates (89% BP). On the postcranial data, australosphenidans diverge from very deep in the tree, outside a weakly supported (35% BP) clade that includes Theriimorpha, Haramiyida, and Docodonta (Figure 4D).

**FIGURE 5:**
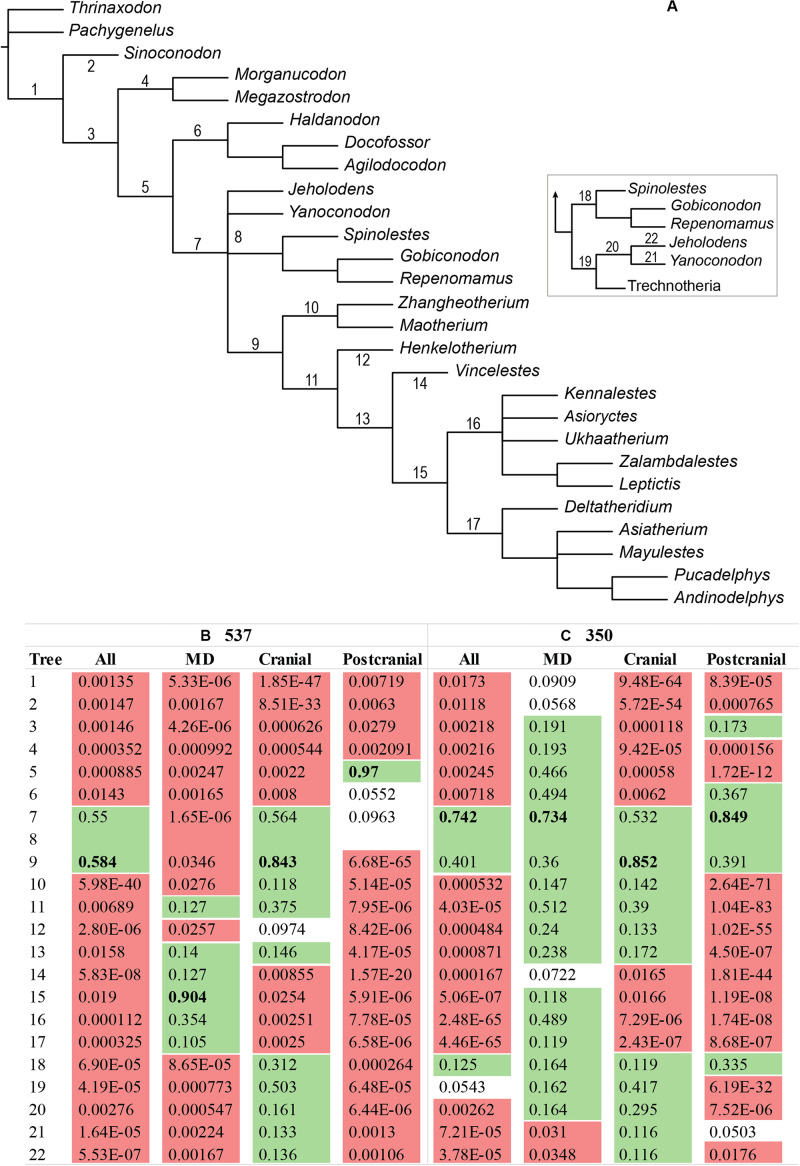
Monotreme placement. Kishino–Hasegawa tests (in IQ-TREE) for the alternative placements of Australosphenida on **(A)** the NCDP mammal backbone constraint phylogeny for the mandibulodental, cranial, and postcranial data for **(B)** the full character set and **(C)** excluding cheek teeth and shoulder girdle characters. The placement of Australosphenida is rejected in red (*P* < 0.05), not rejected in green (*P* > 0.1), and weakly rejected when not highlighted (*P* = 0.05–0.1). Placement 8 corresponds to the placement of Australosphenida within or adjacent to eutriconodonts and is inclusive of placements 7 and 8, and any placements with *Jeholodens* or *Yanoconodon*.

The regional incongruence for the placement of australosphenidans does not depend on the inclusion of the enigmatic haramiyidans and multituberculates. We compared alternative hypotheses for the placement of australosphenidans on the NCDP backbone phylogeny (without multituberculates and haramiyidans). Maximum likelihood analyses on the 537-character matrix (Figure 5B) favored Australosphenida being placed (1) as sister to crown therians on the mandibulodental data, (2) as sister to Trechnotheria on the cranial data and, (3) far deeper on the postcranial data, as sister to Theriimorpha plus docodonts. Notably, the favored placement of Australosphenida as sister to Trechnotheria on the full 537-character matrix is rejected at *P* < 0.05 on both the mandibulodental and the postcranial data.

### Investigating Finer-Scale Regional Homoplasy

We investigated correlated homoplasy induced by the inclusion of the apomorphic clades at a finer anatomical scale among the 537-character matrix. MP disadvantage was calculated for each of the 10 sub-regions as the percentage difference between the MP tree score on that sub-region data compared to the MP score on the same sub-region data, but with the tree constrained to the total evidence topology favored for the overall 537-character dataset.

**FIGURE 6:**
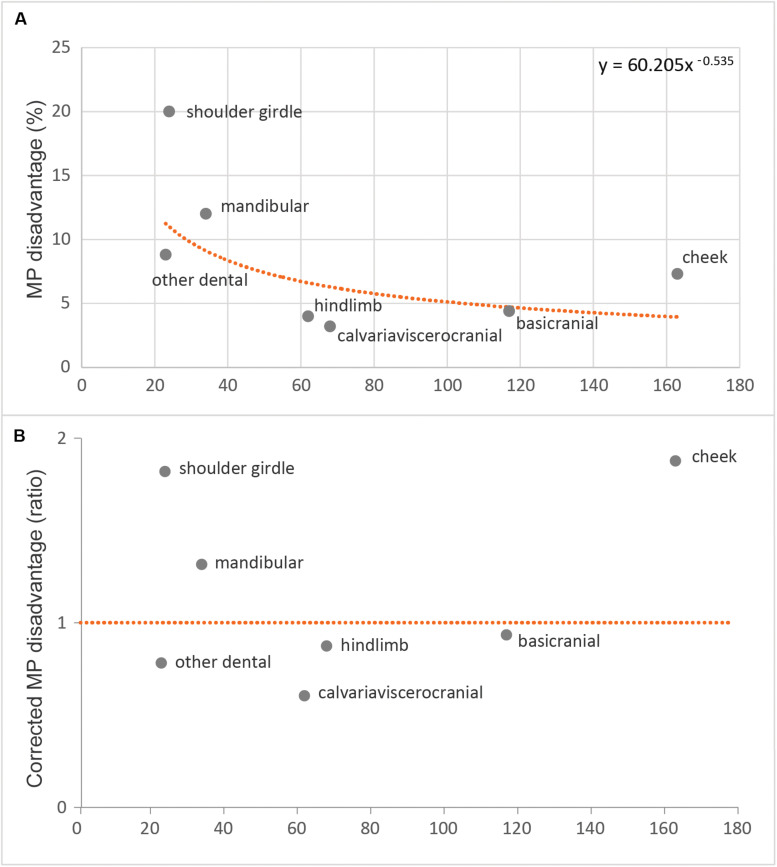
Maximum parsimony disadvantage for each sub-region **(A)** expressed as a percentage and regressed across sub-regions as a power curve and **(B)** corrected maximum parsimony disadvantage for those same values, but compared as a ratio relative to their expected values from the power curve regression.

The highest MP disadvantages were attributable to the shoulder girdle (20.0%) and mandibular (12.0%) sub-regions, and the lowest MP disadvantages were attributable to the basicranial (4.4%) and the hindlimb (4.0%) sub-regions (Figure 6A). A power curve was fitted to control MP disadvantage for the number of characters in each sub-region (Figure 6A). The resulting (inferred/expected) “corrected” MP disadvantage ratios are highest for the shoulder girdle (1.74) and the cheek teeth (1.66) and are lowest for the hindlimb (0.55) and “other dental” (0.75) sub-regions (Figure 6B). All other sub-regions with sufficient character sampling had corrected MP disadvantage ratios between 0.79 and 1.24, close to the expected value of 1. Given this finding, we revisited our primary phylogenetic and incongruence analyses with the cheek teeth and the shoulder girdle characters excluded, leaving a 350-character dataset (51_350_).

### Correlated Homoplasy Reduction

Excluding the cheek teeth and the shoulder girdle characters in the MP analysis of the 51_350_ dataset barely reduced the overall homoplasy index (HI) from 0.52 to 0.49. However, the objective was more specifically to exclude major sources of correlated homoplasy predicted to affect the placements of Australosphenida, Haramiyida, and Multituberculata. Bayesian inference analyses showed a dramatic improvement in congruence among the mandibulodental, cranial, and postcranial region partitions. This is clearly shown in Figure 3, where 95% HPDs for tree likelihood are compared between analyses with topology linked or unlinked (free to vary across partitions). The HPDs are widely separated for the 51_537_ dataset (Figure 3B), indicating a significant incongruence between regional phylogenetic signals. With the cheek teeth and the shoulder girdle characters excluded, the 95% HPDs for tree likelihood are brought into a wide overlap for the 51_350_ dataset (Figure 3C).

Maximum likelihood analyses of the 51_350_ dataset (which excludes the correlated-homoplastic cheek teeth and shoulder-girdle characters) yield notably different results from those of the 51_537_ dataset (Figure 4). For the mandibulodental partition, the multituberculate–haramiyidan and australosphenidan–crown therian groupings recovered with the 51_537_ data (Figure 4B) are both rejected with the 51_350_ data (Figure 4F). The placements recovered with the 51_350_ dataset (Figure 4F) are instead more consistent with analyses of the full skeletal dataset: multituberculates within Theriimorpha and Australosphenida were excluded from Theriimorpha. Exclusion of the shoulder girdle characters also brought the 51_537_ postcranial placement of australosphenidans (previously outside Theriimorpha and Docodonta, Figure 4D) into closer agreement with the overall tree, as sister to Theriiformes (Figure 4H).

With the regional partitions combined (51_350_), but modeled separately, the exclusion of the cheek teeth and the shoulder girdle characters resulted in the same (51_537_) placement of haramiyidans—falling outside of Mammalia (Figures 4A,E). However, 51_350_ provided increased confidence for grouping multituberculates with trechnotheres (BP_ML_ from 90 to 97%; BPP from 0.81 to 0.99). Moreover, the placements of Australosphenida as sister to Theriiformes or Theriimorpha (Theriiformes plus eutriconodonts) are not rejected for either 51_537_ or 51_350_, although the exclusion of the shoulder and the cheek teeth characters shifts support further in favor of excluding australosphenidans from Theriimorpha.

Comparisons of alternative ML placements of australosphenidans alone on the NCDP backbone are particularly instructive for the mandibulodental and the postcranial regions. With the full character set (51_537_), the accepted placements (not rejected at *P* = 0.05) on these two regional datasets are widely separated. In contrast, upon the exclusion of the cheek teeth and the shoulder girdle characters, the (51_350_) mandibulodental and postcranial partitions both favor the same placement for australosphenidans (as sister to Theriimorpha, Figure 5C). This placement of australosphenidans as sister to Theriimorpha was also favored on the combined 51_350_ data and was congruent with the cranial data.

The loss of statistical power for resolving australosphenidan affinities with the mandibulodental data after the cheek teeth characters were excluded (Figure 5C) does not translate as diminished resolution for 51_350_ when the regions are combined. All australosphenidan placements within Trechnotheria or deeper than the theriimorph stem are rejected in IQ-TREE KH-testing at *P* < 0.05 for both the 537- and 350-character datasets. With the addition of multituberculates, an Australosphenida–Multituberculata grouping was rejected initially at *P* = 0.218, but with this result strengthening slightly to *P* = 0.158 following the exclusion of the cheek teeth and the shoulder girdle characters. Rejection of the Allotheria (Haramiyida–Multituberculata) hypothesis is a far stronger result based on the 51_350_ data (*P* = 0.001) compared with 51_537_ data (*P* = 0.044).

### Extension to Less-Complete Taxa

**FIGURE 7:**
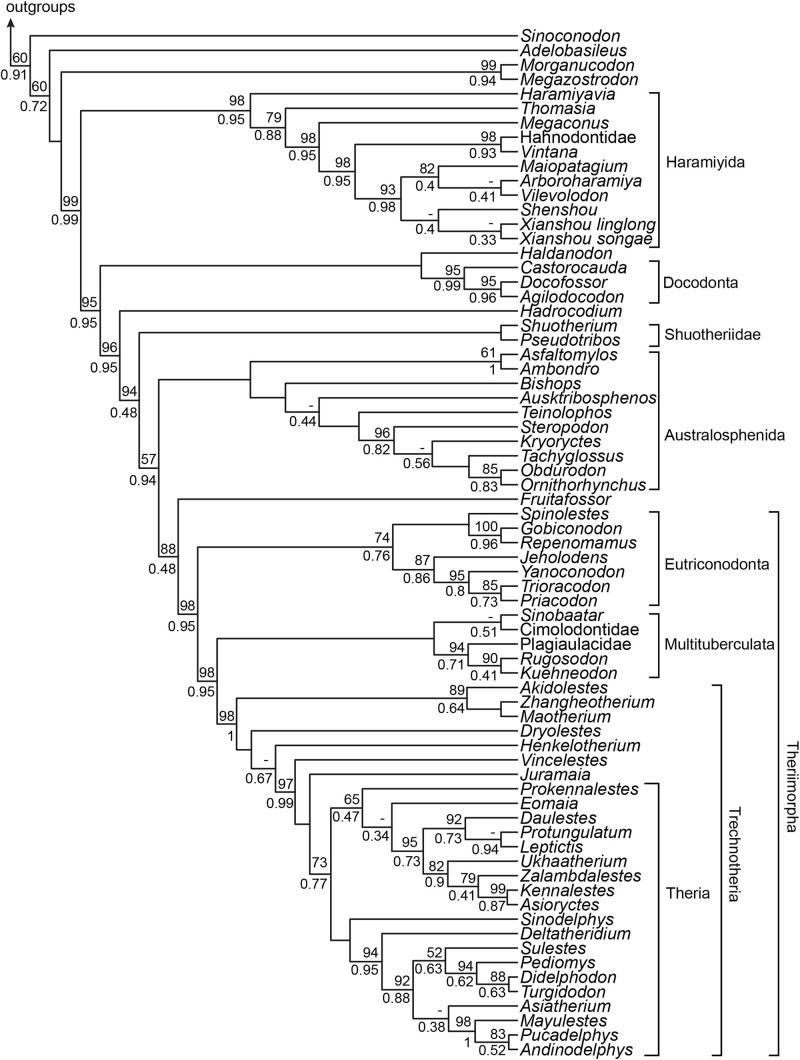
Bayesian tree based on the 78_350_ dataset. The Bayesian posterior probability (BPP) and maximum likelihood bootstrap (BP_ML_) are represented only at nodes where BPP < 1 and BP_ML_ < 100%. The dashes represent branches which are not supported in the maximum likelihood analysis.

Character incompleteness within the more taxonomically inclusive 78_350_ dataset invalidates the further examination of incongruence among regions. However, the increased taxon sampling confirms and generally enhances statistical support for the placements of australosphenidans, multituberculates, and haramiyidans when compared with the 51_350_ dataset (Figure 7, cf. Figure 4E) and recovers a broadly similar topology to that of Huttenlocker et al. (2018). The additional taxon sampling and exclusion of the sub-regions contributing disproportionately high levels of correlated homoplasy also strengthened the support for grouping Hahnodontidae and the Gondwanathere, *Vintana*, as well as for their position within Haramiyida as sister to Eleutherodontidae (98% BP_ML_ and 0.95 BPP).

The 78_350_ data (Figure 7) allows us to examine the phylogenetic implications of excluding the cheek teeth and the shoulder girdle characters for several less complete taxa that are nevertheless important for reconstructing mammalian evolution. Of particular note, the oldest proposed eutherian, *Juramaia* (Luo et al., 2011), was excluded from crown Theria (73% BP_ML_ and 0.77 BPP). The proposed sister taxon of Australosphenida, the Shuotheriidae [see Luo et al. (2007)], was instead placed as sister to Mammalia, although the group composed of Australosphenida, *Fruitafossor*, and Theriimorpha, which define Mammalia in this case, is not confidently resolved (57% BP_ML_ and 0.94 BPP). Analyses on this extended 78_350_ dataset also retrieved a stronger support for placing Australosphenida (and *Fruitafossor*) outside of Theriimorpha (98% BP_ML_ and 0.95 BPP) and for eutriconodont monophyly (74% BP_ML_ and 0.76 BPP).

## Discussion

### Phylogenetic Incongruence Between Anatomical Regions for Ecologically Apomorphic Groups

Complex organisms are able to efficiently evolve through fitness landscapes because developmental modularity results in correlated evolution among characters (Lande and Arnold, 1983; Wagner, 1996; Kemp, 2007). Characters can be functionally and/or developmentally linked by genetic pleiotropy, heterochrony (*e.g*., paedomorphosis), allometric lines of least resistance, and adaptive covariance. The linked characters are therefore unlikely to evolve fully independently. Such evolutionarily correlated character complexes tend to inflate both true phylogenetic signals (*i*.*e*., due to shared ancestry) and non-phylogenetic signals (homoplasy). However, taxa that are more developmentally or functionally divergent are of course more likely to express these correlations as non-phylogenetic signals. Analogous phylogenetic biases are well studied for molecular data, for example, with DNA base composition (Sueoka, 1995; Phillips and Penny, 2003; Gibson et al., 2004), but this issue remains underexplored with morphology. However, from this theoretical expectation, we hypothesized that monotremes and the dietarily apomorphic (omnivorous–herbivorous) multituberculates and haramiyidans would contribute more substantially to phylogenetic incongruence among anatomical regions than the generalized insectivore/carnivore mammalian backbone phylogeny. As anticipated, there is broad phylogenetic agreement between mandibulodental, cranial, and postcranial partitions among the generalized insectivore/carnivores, but the inclusion of ecologically apomorphic clades of australosphenidans (including monotremes), multituberculates, and haramiyidans leads to a significant incongruence in ML hypothesis testing (Table 1) and a significant likelihood advantage for unlinking topologies for anatomical region partitions in Bayesian inference (Figure 3B).

Three regional relationships in Figure 4 stand out as being several tree steps distant from their full-skeletal affinities. These are the mandibulodental groupings of (1) haramiyidans with multituberculates (Allotheria) and (2) australosphenidans with crown therians, as well as (3) the deep placement of australosphenidans on the postcranial data. While previous research has not quantified the incongruence induced by ecologically apomorphic Mesozoic mammals, the patterns are consistent with previous observations. The potential for dental and jaw geometry convergence between the multicuspate, omnivorous–herbivorous “allotherians” has been robustly discussed (*e*.*g*., Jenkins et al., 1997; Butler, 2000; Zhou et al., 2013; Zheng et al., 2013; Bi et al., 2014; Luo et al., 2015). Also, early suggestions of close homology between the molar morphology of australosphenidans (or stem monotremes) and therians (*e*.*g*., Archer et al., 1985; Rich et al., 1997) are less probable in view of more recent fossil finds, and the independent evolution of near-tribosphenic molars in therians and australosphenidans is well founded in form/function cusp pattern models (*e*.*g*., Luo et al., 2001; Rougier et al., 2007; Davis, 2011).

Postcranial character complexes can have homoplasies (*e*.*g*., Ji et al., 1999, Meng et al., 2017), and widespread postcranial homosplasy has been suggested for monotremes (Gregory, 1947). Shoulder girdle morphology and some upper appendicular features of monotremes appear to be superficially similar to those of ancient cynodonts (*e*.*g*., Macintyre, 1967; Gambaryan et al., 2015). Such similarities are homoplastic when mapped on phylogeny as monotremes (and other crown mammals) are widely separated from non-mammaliaform cynodonts, according to modern phylogenetic interpretations that consider a wide range of new Mesozoic mammaliaforms that were unknown 50 years ago (Figure 4). The historical argument for monotremes to be related to some cynodonts (Macintyre, 1967) is also inconsistent with relaxed-clock molecular dating for the divergence of monotremes and therians (*e*.*g*., Kullberg et al., 2008; Meredith et al., 2011; Phillips, 2015). Developmental studies on the monotreme and the marsupial shoulder girdles (*e*.*g*., Klima, 1973) leave open the possibility that the monotreme condition is partly paedomorphic, while the extreme humeral long-axis rotation emphasis in monotremes associated with fossorial/swimming activity offers functional arguments for an evolutionary reversal upon earlier cynodont conditions (Phillips et al., 2009). In addition, the *Kryoryctes* humerus (Pridmore et al., 2005) suggests reversal from trochlea-like to condylar ulnar articulation. A somewhat similar transformation (at least with a shallow trochlea condition) may also have occurred in marsupial moles [noting the Miocene and modern species figured in Archer et al., 2011)]. Developmental genomics may offer a pathway to testing the hypotheses of homology *versus* homoplasy involving monotremes and other extant mammals (or reptiles), as has been done for female reproductive organs (Wagner and Lynch, 2005).

It is encouraging that our MP disadvantage analyses identified the same two anatomical character complexes (cheek teeth and shoulder girdle) that had prior expectations (*e*.*g*., Kangas et al., 2004; Phillips et al., 2009; Ramírez-Chaves et al., 2016) for substantial correlated homoplasy upon the inclusion of australosphenidans, multituberculates, and haramiyidans with the generalized insectivore/carnivores. It is not obvious from standard homoplasy metrics, such as the HI, that either the cheek teeth or the shoulder girdle is especially problematic; of the 10 sub-regions, they are ranked only fifth and seventh for HI (Supplementary Table S3). HI may be a poorer indicator of phylogenetic inaccuracy. If homoplastic transformations are randomly distributed, then increasing homoplasy may reduce phylogenetic resolution but may not manifest as statistical inconsistency. MP disadvantage instead considers the difference between MP scores on a given partition, for when the tree is unconstrained, compared to when the tree is constrained to the topology inferred from all partitions. The more phylogenetically correlated the homoplasy, the larger the MP disadvantage. Beyond the present study, it will be important to further explore the relationship between MP disadvantage and the relative size of the partition to the overall dataset (which we correct for in Figure 6B). Resampling procedures may also permit confidence intervals for MP disadvantage.

### Mesozoic Mammal Phylogeny and the Constituency of the Crown Group

The relationships between the major groups of generalized insectivore/carnivores that were included in the near-completely sampled, dietarily plesiomorphic dataset (NCDP_537_) are well established (*e*.*g*., Kielan-Jaworowska et al., 2004; Rougier et al., 2011; Martin et al., 2015). Here we confirm strong support for their branching order, from non-mammaliaform cynodonts right through to eutherians and metatherians (Figure 2A)—with or without the cheek teeth and the shoulder girdle characters and with extended taxon sampling (Figure 7). However, by investigating phylogenetic signal among broad-scale anatomical regions (Figures 4A–D), we show that the affinities of three dietarily apomorphic clades, multituberculates, haramiyidans, and australosphenidans (including monotremes), are less stable than total evidence approaches might imply.

There is a common argument that combining all relevant evidences provides the best phylogenetic estimator (Asher, 2007; Mounce et al., 2016). Similar to advocacy for parsimony over likelihood, this simpler solution may well be appropriate when underlying evolutionary processes are largely unknown (Kolaczkowski and Thornton, 2004). However, our interrogation of phylogenetic signal variation across partitions reveals extreme incongruence (Figures 3B, 4B–D, 5). To ignore phylogenetic incongruence in favor of the total evidence approach to combine the incongruent data partitions is akin to ignoring interaction effects in a statistical analysis of variance. Instead we have identified elevated levels of correlated homoplasy among the cheek teeth and the shoulder girdle (Figure 6). Excluding these data was remarkably effective for bringing a phylogenetic congruence between the mandibulodental, cranial, and postcranial partitions (Figures 3C, 4F–H, 5). Moreover, reducing the overall dataset from 537 to 350 characters did not cost precision. In fact, in some cases, phylogenetic resolution was greatly enhanced, such as for rejecting Allotheria (Multituberculata–Haramiyida) at *P* = 0.001 (51_350_) compared with *P* = 0.044 (51_537_). This strong result in favor of multituberculates grouping with trechnotheres and haramiyidans falling outside crown Mammalia was foreshadowed by the analysis of Ramírez-Chaves et al. (2016), with molar characters excluded. A note of caution is nevertheless warranted for the relative placements of these multicuspate taxa. Even beyond arguments for and against dental convergence, there is substantial variation and debate regarding non-dental characters among haramiyidans and multituberculates (Bi et al., 2014; Luo et al., 2015; Meng et al., 2018). Resolving character scoring and including further cranial material from undoubted haramiyidans will be important for confirming our placement of multituberculates within and haramiyidans outside Mammalia.

Our efforts to identify the affinities of Australosphenida are broadly reflective of most matrix-based analyses over the past decade, which tend to place this Gondwanan clade either as the sister of Theriiformes (trechnotheres and multituberculates) or a further step stemward, also outside of eutriconodonts. Our incongruence findings do reveal instability in this near-consensus, and an Australosphenida–Multituberculata clade is not yet convincingly rejected; however, there is progress toward resolution. In particular, exclusion of the sources of substantial correlated homoplasy (Figures 4, 5) and expanded taxon sampling (Figure 7) both strengthen the support for australosphenidans falling outside of Theriimorpha. Thus, trechnotheres, multituberculates, and eutriconodonts would all be crown mammals. The case is weaker for the somewhat fossorial and dentally simplified *Fruitafossor* (Luo and Wible, 2005) and for the pseudotribosphenic shuotheriids (Figure 7). Discovery of substantial skeletal material from *Pseudotribos* (Luo et al., 2007) added weight to the earlier hypothesis of Kielan-Jaworowska et al. (2002) that shuotheriids were the sister group of australosphenidans. The primary arguments were based on tentative interpretations of molar morphology and seemingly more robust (although ambiguous) shoulder girdle synapomorphies. These are notably the same character complexes that our MP disadvantage analysis identified as the most likely to be unreliable for inferring the affinities of highly apomorphic Mesozoic mammals. Hence, we suggest that placement of shuotheriids with australosphenidans (or even within Mammalia) requires further testing, ideally on more complete cranial and postcranial material.

On balance our incongruence testing, identification of sources of elevated correlated homoplasy, and partitioned ML and Bayesian phylogenetic inference offer increased confidence in the relationships of the main Mesozoic clades of generalized insectivorous/carnivorous mammals and stronger support (albeit with caveats) for the placements of australosphenidans, multituberculates, and haramiyidans. We also identify where additional caution is required, for example, we join several other studies (Krause et al., 2014; Averianov, 2015; Sweetman et al., 2017) in questioning the placement of *Juramaia* as a eutherian. The implication for molecular dating is that more completely known crown therians from the ∼125 Ma Jehol biota may provide a safer Early Cretaceous minimum bound for calibrating the marsupial–placental divergence than does the purportedly Jurassic *Juramaia*. Current progress toward improved inference of phylogeny and dating with appropriate calibrations (Phillips and Fruciano, 2018) present an opportunity to more accurately trace the ecological ancestry of early mammals. This, in turn, can shed light on the biotic and abiotic drivers of convergent dietary and locomotory evolution among Mesozoic mammals.

## Data Availability Statement

The datasets generated for this study are available on request to the corresponding authors.

## Author Contributions

MP conceived the study. MC and MP compiled and analyzed the dataset and wrote the manuscript. All authors contributed to the article and approved the submitted version.

## Conflict of Interest

The authors declare that the research was conducted in the absence of any commercial or financial relationships that could be construed as a potential conflict of interest.
