# Supplementary material for: Conflict Resolution for Mesozoic Mammals: Reconciling Phylogenetic Incongruence Among Anatomical Regions
Source: Front Genet. 2020 Jul 8;11:0651. doi: 10.3389/fgene.2020.00651 (PMC7381353; doi:10.3389/fgene.2020.00651)
Supplement: Supplementary file 1 [file Data_Sheet_1.docx]

***Supplementary Material***

**Conflict resolution for Mesozoic mammals: reconciling phylogenetic incongruence among anatomical regions**

Mélina A. Celik^1*^, Matthew J. Phillips^1*^

^1^*School of Biology and Environmental Science, Queensland University of* *Technology, Brisbane 4000, QLD, Australia.*

**Content**

1. Morphological matrix
2. Initial exploratory analyses
3. Testing phylogenetic congruence between anatomical regions
4. Homoplasy within anatomical regions
5. Correlated homoplasy reduction and extension to less complete taxa
6. **Morphological matrix**

The character matrix is derived from Huttenlocker et al. (2018), which was primarily built by Zhou et al. (2013), Bi et al. (2014), and Luo et al. (2015a, b). The original matrix of Huttenlocker et al. (2018) (Hutt) covers 538 morphological characters of 125 mammal and non-mammalian synapsid taxa. Characters include osteological (mandibular, dental, cranial, and postcranial) and soft-tissue characters. We have excluded soft-tissue characters (characters 511-513), modified scores of some mandibular characters for some Australosphenida taxa, included additional characters, and we have included the stem monotreme species *Kryoryctes cadburyi* (Pridmore et al. 2005). Modifications are indicated below.

***a) Revised character scoring***

Character 1: Post-dentary trough

*Steropodon* (Hutt: 0, here: ?). From Ramírez-Chaves et al. (2016), “Comprehensive reviews now tend to support the absence of a postdentary trough from both species [18, 29, 30].” That refers to *Teinolophos* and *Steropodon*. Howerer, it is hard to interpret the broken/missing portion of *Steropodon* and so this determination is not yet sufficiently well founded. Therefore Ramírez-Chaves et al. (2016) favoured uncertainty (?). We concur.

*Ausktribosphenos* and *Bishops* (Hutt:0, Us:2). This follows Ramírez-Chaves et al. (2016), also Rougier et al. (2011) and Rich et al. (2016).

Character 2: Separate scars for surrangular/prearticular in the mandible

*Ausktribosphenos* (Hutt: 0, here:1). We assign the same state as Hutt gave *Bishops*. For *Ausktribosphenos* this follows discussions in the same references as cited for character 1.

*Steropodon* as 1 (absent) is appropriate – although it is hard to tell if it’s even a PDT, there is no clear evidence for separate scars.

Character 4: Degree of development of Meckel’s sulcus.

*Steropodon* (Hutt: 0, here: ?). See discussion in Ramírez-Chaves et al. (2016). Hard to distinguish from damage.

Character 5: Curvature of Meckel’s sulcus

*Steropodon* (Hutt: 0, here: ?). See discussion in Ramírez-Chaves et al. (2016). Hard to distinguish from damage.

Character 12: Coronoid bone.

*Teinolophos* (Hutt:2, here: ?). Instead of no coronoid bone, Rich et al. (2016) indicate possible coronoid bone contact area.

Character 13: Location of the mandibular foramen.

*Steropodon* (Hutt: 0, here: ?). Again, is hard to say anything conclusive given damage etc.

Somewhat surprising that the state is 0 (within PDT or posterior Meckel’s sulcus) in *Ornithorhynchus*, which does not possess either – but this could be justified by Ramírez-Chaves et al. (2016) Fig 3, looking at the juvenile. Similar for *Obdurodon* we would expect.

***b) Additional characters***

Character 539: From Phillips et al., 2009. Adult body size, (0) <500g; (1) ≥ 500

Character 540: From Phillips et al., 2009. Mandibular aspect ratio for most of the length of the dentary below the tooth row, or homologous section (0) depth at least equal to width; (1) depth less than width.

***c) Scoring of* Kryoryctes *humerus***

Character 263. Intertubercular groove of the humerus: (0) Shallow and broad; (1) Narrow and deep.

*Kryoryctes* = 0

Character 264. Size of the lesser tubercle of the humerus relative to the greater tubercle: (0) Wider; (1) Narrower.

*Kryoryctes* = 0

Character 265. Torsion between the proximal and distal ends of the humerus: (0) Strong (≥30 degrees); (1) Moderate (30–15 degrees); (2) Weak.

*Kryoryctes* = 0

Character 266. Ventral extension of the deltopectoral crest or the position of the deltoid tuberosity: (0)

Short and limited to the proximal part of the humeral shaft; (1) Extending ventrally (distally) at least 1/3 the length of the shaft.

*Kryoryctes* = 1

Character 267. Teres tuberosity on medial side of humerus. (0) Absent; (1) Present; (2) Hypertrophied.

*Kryoryctes* = 2

Character 268. Ulnar articulation on the distal humerus: (0) Bulbous ulnar condyle; (1) Cylindrical trochlea in posterior view with a vestigial ulnar condyle in anterior view; (2) Cylindrical trochlea without an ulnar condyle (cylindrical trochlea extending to the anterior/ventral side).

*Kryoryctes* = {12} No evidence for a vestigial unlar condyle, but that breakage may mean it was not preserved, but even so, as Pridmore et al. (2005) note, if it was there it would be offset somewhat from the olecranon fossa, so would have to be quite vesitgial in a functional sense if there at all.

However, IQTREE cannot use uncertain/polymorphic characters, and 268 is a key character. So although we employ the most conservative coding {1,2} for maximum parsimony analyses, we use state 1 for the analyses in IQTREE (and for comparability, in MrBayes). This is because, while it is unknown whether there is a vestigial ulna condyle, the trochlea does not extend ventrally, so on that basis and certainly in the “spirit” of the character definition, the condition in *Kryoryctes* is intermediate (i.e. state 1).

Character 269. Radial articulation on the distal humerus: (0) Distinct and rounded radial condyle in both

anterior (ventral) and posterior (dorsal) aspects (that does not form a continuous synovial surface with the ulnar articulation in the ventral/anterior view of the humerus); (1) Rounded radial condyle anteriorly but cylindrical posteriorly; (2) Capitulum (forming a continuous synovial surface with the ulnar trochlea; cylindrical in both anterior and posterior aspects).

*Kryoryctes* = 0

Character 270. Entepicondyle and ectepicondyle of the humerus: (0) Robust; (1) Weak.

*Kryoryctes* = 0

Character 271. Sigmoidal shelf for the supinator ridge extending proximally from the ectepicondyle: (0) Absent; (1) Present

*Kryoryctes* = 0

***d) Data partitioning***

Morphological characters were divided into partitions based on three anatomical regions: mandibulodental (220 characters), cranial (183 characters), and postcranial (134 characters). Characters were also assigned to 10 finer partitions based on the following sub-regions: cheek teeth, dental, mandibular, basicranial, calvariaviscerocranial, axial, shoulder girdle, forelimb, pelvic girdle, and hindlimb

Regions

charset mandibulodental = 6-16 19-221 514 515 519 520 537 540;

charset cranial = 1-5 17 18 355-510 516-518 521-536 539;

charset postcranial = 222-354 538;

Sub-regions

charset mandibular = 6-16 19-40 540;

charset cheek teeth = 41-175 190-198 203 206-216 218-221 514 515 537;

charset other dental = 176-189 199-202 204-205 217 519 520;

charset basicranial = 1-5 17 18 355-452 521-532;

charset calvariaviscerocranial = 453-510 516-518 521 533-536 356 539;

charset axial = 222-237;

charset shoulder_girdle = 238-261;

charset forelimb_manus = 262-278;

charset pelvic_girdle = 279-291;

charset hindlimb_pes = 292-350 352-353 538;

1111111111222222222233333333334444444444555555555566

Taxon/Node 1234567890123456789012345678901234567890123456789012345678901

-------------------------------------------------------------------------------

Thrinaxodon 000000000000000000000000000-0-0-0-010000------------0-0-00000

Massetognathus 000001000000000001000000000-0-0-00010000------------0---20000

Probainognathus 000000000000000001000000000-000-00010000------------0---00000

Tritylodontids 000001000000000000000000010-000-30002000------------10--20-00

Pachygenelus 0000000000000100000000000010000-20000100-----0------1-1-00010

Brasilitherium 00-000000-00---00---01100---00100001010000----------0-0100000

Brasilodon 0--000000-0-----0---011000--00100001010000---------00--100000

Adelobasileus -------------------------------------------------------------

Sinoconodon 00000010000000000000000000110100000001000--00------00-1100011

Morganucodon 00001010000000000000000000200100000001000--00-0000001-0100011

1

Megazostrodon 00001000000000000000000000200200100001000--00-0000-01-0000011

Haldanodon 00000110001000100010000000210100100001000--00-0000000-0100011

Castorocauda 00-00110001---1-0000000000210100100011000--00-0---0----100011

Docofossor 00000100000100110-000000002101003000110000-10-00-000--0100011

Agilodocodon 000001100011001100000000002101002000110000-10-000000--0100011

Megaconus 10000-100000001-000000000-220100-0002100100000-----011--2-011

Cifelliodon --------------------------2----------------------------------

Millsodon -------------------------------------------------------------

Hahnodon -------------------------------------------------------------

Eleutherodon ----------------------------------------1----0--------------1

Sineleutherus ----------------------------------------111-1-------21--2-0-1

Thomasia -----------------------------------------00001-----0-0--0--00

Haramiyavia 0-0001000000000-0-0-00000001000000000100-------------0--0-000

Arboroharamiya 1111-13000012021-01111000022030010002101111210------21--23300

Xianshou linglon 111--13000012021-01121000022030010002101111210-----021--23300

Xianshou songae 1112-130000-2021-01121000022030010002101111210-----021--23300

Shenshou 1111-13000012021-01121000022030010002101111100-----020--22200

1

Vilevolodon 1111-130000-2021101111000022030010002101111210-----021---3300

Maiopatagium -------------------------------------------100-----02--------

Vintana -------------------------------------------------------------

Rugosodon 2112-10--00-302-10102120102224001000210110001------030--2-000

Kuehneodon 2112-10--0013021101021201022240010002101100011-----030--2-000

Sinobaatar 2112-10--0023021101021201022240010002101100010-----030--2-000

Plagiaulacids 2112-10--0013021101021201022240010002101100011-----030--2-000

2

Cimolodontans 2112-10--0023021101021201022140010002101100000-----030--2-000

2 12 1

Hadrocodium 0--2-130010-40211000000000200100200-01000--00-0000101-1000011

Shuotherium 0010110--0000021--000-00-0----0-000101000-----------0-1111100

Pseudotribos 0-10011000-0001---00------210101000111000------0---00-1111100

Asfaltomylos 0012112110000021--0022000021310120010--00-------------11011-0

Ambondro -----1---00----1--------0-----0---0-0--00-------------12131-0

Ausktribosphenos 2110012010000021--002200002--101200101000--00---------1213100

Bishops 21-10120100-0021--0022000021310120010-000--00---------1213100

Teinolophos 21002120100?3021--0022000-21310120010--00--00---------------0

Kryoryctes -------------------------------------------------------------

Steropodon ?11??1---0--?0-1-------------------10------00---------------0

Obdurodon 21-2-1201002302110112200012131112111010-0--00---------1013100

Ornithorhynchus 21-2-100100230211011220001213111211101000--00----------------

2

Tachyglossus 21-2-10--00240211000000000213111--1-01000--00----------------

Fruitafossor 21-0014000020021--110000112101011001-1000--00-0000000-10-0010

Gobiconodon 21-1010--00110211012210011211400100101000--00-0000001-1101001

2

Repenomamus 21-1010--00210211012210011211400100101010--00-0000001-1101011

Spinolestes 21-0010--00210211012210011221401-0-1010000--0-0000000---00010

Amphilestes 21-11-0--002102---122--0--211400200201-00--00---------11010-1

Yanoconodon 21-1010--0021021101221001-221400100001000--00-0000001-0000011

Jeholodens 21-1010--00-10211012--001-221400100001000--00-0000001-0101011

Trioracodon 21-10-0--0021021--12210010221400100001000--00-00000-1-0101011

Priacodon 21-1010--0021021--12210010221400100001000--00-0000001-0001011

Tinodon 2--11-----001021--121-00---104-130-00--00-------------01010-0

1

Akidolestes 2----10--00-20----12210010211401100111000--00-00000-1-1101000

Spalacotherium 2111010--001202-----210010211401100111000--00-00000-1-1101000

Zhangheotherium 21-0010--0012020--1221001-211401010111000--00-0000011-1101000

Maotherium 21-0010--0012020--12210010211401010111000--00-0000011-1101000

Dryolestes 21-1011100012020--11210-01211301200111000--00-0000011-0000011

Henkelotherium 21-1011100012021--112101--211301200111000--00-00000-1-0000011

Amphitherium 21-1111100012021--11210-01210301200111000-----11-0-0--00000--

Peramus 21-1111100012021--11210101210301200111000--00-1100201-0002011

Vincelestes 21-2-11100012021--11110101211301200111000--00-0000101-1000000

2

Nanolestes 21-1011100012021--112-0--1210301200111000-------------00000-1

Kielantherium 21-101110-012-21--111101-2----0130011-000-------------00000-1

Aegialodon ---------------------------------------00--------------------

Montanalestes 21-2-13100014021--11120103210301300111000-------------12020-1

Prokennalestes 21-1113100011021--11120102210301200111000--00-10112-1-1202001

1

Murtoilestes ----------------------------------------0--------------------

Eomaia 21-1113100011021--11120102210301200111000--00-1011111-1201001

Juramaia 2-12--11000---------1201022-0301100111000--00-1011201-1201001

Kennalestes 21-2-1310001402110111200022---0-200111000--00-1111201-1002001

Asioryctes 21-2-131000140211011120002210301300111000--00-1112201-1102001

Ukhaatherium -1-------00-40----------022---0--00-11000--00-1112201-1102001

Zalambdalestes 21-2-111000140211011120002210301200111000--00-1112211-0212201

3 2 1

Daulestes 21-2-131000240211011120002210301200111000--------1--1------01

Aspanlestes -----1----0--0---------1------0---0-1-0-0--00-11112-1-1-010-1

Eoungulatum -----1-----------------1------0---0-1---0--00-10110-1-------1

Cimolestes 2--2-1010-0240211-111200-22103012-0111000--00-10122-1-1101001

Gypsonictops 2--2-101000240211-111200-22103012-0111000--00-11122-1-1212001

Protungulatum 21-2-101000240211-11120002210301200111000--00-10120-1-1211001

Erinaceus 21-2-131000241211011120002210301300111000--00-1011011-1212200

Leptictis 21-2-131000240211011120002210301300111000--00-1112211-1212200

Canis 211--131010240211011110002211301300111000--00-0100011-0011010

Felis 211--131010240211011110002211301300111010--00-0100011-0011010

Rattus 2112-111000241211011112000222-01100021010-----------0------00

Oryctolagus 2112-111000240211011111000222-011-1121010--00-0012000-0010000

Bradypus 211--1310102412110111100022113013-0111000-----------0------00

Tamandua 211--1310102412110------0-21-30---1--10-0--------------------

Glyptotherium 211--131010241211011110002211301310111100-----------0------00

Dasypus 211--131010241211011110002211301310111100-----------0------00

Chaetophractus 211--131010241211011110002211301310111100-----------0------00

Euphractus 21---131010241211011110002211301310111100-----------0------00

Holoclemensia ----------------------------------0-1---0------------------11

Sinodelphys 21---131010-4021--111-0--2210301300111000--00-00--111--101011

Deltatheridium 21-2-14101022021--11110004210301300111000--00-0000111-0001011

Atokatheridium -----------2----------------------0--------00----------------

Sulestes 21-2014101022021--111100142-1-01310111000--00-000011--0001011

Asiatherium 21-1-141010240211-111100142-0-01300111000--00-0000111-0001011

Kokopellia -1-1-1410-022--1--1-1100-32-----3-0111000--00-----1---0001011

4

Anchistodelphys ----------------------------------0--------00---------------1

Albertatherium ----------------------------------0--------00---------------1

Didelphodon 21-2-14101021021--11110014211301-00111000--00-001---1-0001101

Pediomys ---2-14101021021--11110014211301-00111000--00-------1-------1

Turgidodon ---2-141010210211-111100-4-11301-00111000--00-------1-00010-1

Mayulestes 21-2-14101024-211011110014210301300111000--00-0000111-0001011

Pucadelphys 21-2-141010240211011110014210301300111000--00-0000111-0001011

Andinodelphys 21-2-141010240211011110014210301300111000--00-0000111-0001011

Didelphis 21-2-141010241211011110014210301300111000--00-0000211-0001011

Marmosa 21-2-141010241211011110014210301300111000--00-0000211-0001011

Caenolestes 21---14101024-211011110014210301300111000--00-------1------01

Dasyurus 21-2-141010240211011110014210301300111000--00-0000211-0001011

1

Perameles 21---141010241211011110014210301300111000--00--------------00

Dromiciops 2112-141010240211011110014210301300111000--00-000021--0001000

Thylacomyidae 211--14101024-211011110014210301300111000--00-------1------10

Macropus 211--141010240211011110014210301300111000--00--------------00

Acrobates 211--141010241211011110014210301300111000--00--------------00

Phascolarctos 211--131010240211011110014210301300110000--00--------------00

Vombatus 211--141010240211011110014210301300110000--00--------------00

Phalanger 211--141010240211011110014210301300111000--00--------------00

Pseudocheirus 211--141010240211011110014210301300111000--00--------------00

Petauroides 211--141010240211011110014210301300111000--00--------------0-

Input data matrix (continued):

11111111111111111111111

6666666677777777778888888888999999999900000000001111111111222

Taxon/Node 2345678901234567890123456789012345678901234567890123456789012

-------------------------------------------------------------------------------

Thrinaxodon 0000---------00----0000----------0000-000---0----------------

Massetognathus 0000---------20----0400----------0000-000--------------------

Probainognathus 0000---------00----0000----------0000-000---0----------------

Tritylodontids 0000--------021----24000-00------0000-000--------------------

Pachygenelus 0000---------00----0000----------0000-000--000---------------

Brasilitherium 020---0------00----0-00---------------000---00---00----------

Brasilodon 020---0------00----0-00----------2----000---00---00----------

Adelobasileus -------------0------000----------0000-000--000---------------

Sinoconodon 0000-00-0----00----0000------20--01000000-1000---0000---0----

Morganucodon 0200-00-0----00-0--1100------20--2100000110000---0000---0----

Megazostrodon 0200-00-0-0--00-0--1200------20--2100000100000---0000---0----

Haldanodon 020101010-0--00-0--1100------20--21031200-0000---0000---0----

Castorocauda 020101000----00-0--1100------2---20031200-0000---0000---0----

Docofossor 020101000----00-0--1100-----020--21031200-0000---0000---0----

Agilodocodon 021112010----00-0--1100-----000--21031200-0000---0000---0----

Megaconus 2201--300000221----240100011-----0--00-0122------------------

Cifelliodon ---------10---1----------------------------------------------

Millsodon ---------111--1----------------------------------------------

Hahnodon ---------10---1----------------------------------------------

Eleutherodon 20-------11122-----241121120-----0--0--0120------------------

Sineleutherus 20-0---01----21----241120100-----0--0--0120------------------

2

Thomasia -000---10100021----240110100-----0--0--0---------------------

Haramiyavia 0000--200100021----240110100-----0--0--0120------------------

Arboroharamiya 1000--3--210221----241121120-----0--0--01-2------------------

Xianshou linglon 1000--3--111021----241120100-----0--0--01-0------------------

Xianshou songae 1000--3--111021----241120100-----0--0--01-0------------------

Shenshou 2000--3--110021----241111000-----0--0--01-0------------------

Vilevolodon 1020-----211221----241121120-----0-00--00-0------------------

Maiopatagium ---------000021----24----------------------------------------

Vintana ------------------0-5----------------------------------------

Rugosodon 01-0----1000121----240111000-----0--0--00-0------------------

Kuehneodon 0120--311000021----240101000-----0--0--00-0------------------

Sinobaatar 0010--201000121----240101001-----0--0--00-0------------------

Plagiaulacids 0120--211000121----240101000-----0--0--00-0------------------

1 1

Cimolodontans 0000--200000121----240100010-----0--0--00-0------------------

2 1 21

Hadrocodium 0000-0--0----00-0--1200------20---0000000-0000---0000---0----

Shuotherium 0011--0-0----13-2001200-----0100-13230210-01110000100---0----

Pseudotribos 00-1110-0----13-2001200-----0100-13230210-0111000-100---0----

Asfaltomylos --------0----13-2--1300-------13-02100000--111022141100011112

Ambondro --110---0----13-2--1300-----0-13300120100--111022241120-11112

Ausktribosphenos 0011011-0----13-2--1300-----0213300120100-0221022141220032123

Bishops 00-1011-0----13-2--1300-----0213300120100-1221022141220032123

Teinolophos --------------3-2--1300-----1111200120010--222042231210-02-23

Kryoryctes -------------------------------------------------------------

Steropodon --------0----13-2--1300-----1111200120110-0222042231210-02-23

Obdurodon --00----0----13-2--1300-----1111200120010-0222042231210-02-23

Ornithorhynchus -------------13-2--1-00-----1-1-----2-010-0222042231210--2-23

Tachyglossus ---------------------00--------------------------------------

Fruitafossor 0000-0--0----------1000----------0--00000-0------------------

Gobiconodon 0010000-0----00-0--1200-----010--1210000100-00---0000---0----

Repenomamus 0010000-0----00-0--1200-----010--1210000100-00----000---0----

Spinolestes 0-----0-0----00-0--1200-----010--000000013--00---0000---0----

Amphilestes 0000000-0----00-0--1200-----010--1010000100-00---0000---0----

Yanoconodon 0000000-0----00-0--1100-----010--0100000121-00----000---0----

Jeholodens 0000000-0----00-0--1100-----010--0100000121-00---0000---0----

Trioracodon 0000100-0----00-0--1100-----110--1000000121-00---0000---0----

2

Priacodon 0010100-0----00-0--1100-----110--1200000121-00---0000---0----

Tinodon 0000-00-0----1201--1200------10001010000100-110000100---00--0

Akidolestes 0000120-1----1302011200-----01000100000013012200-0100---00--0

Spalacotherium 0000100-1----1312001200-----01000100000013011200-0100---00--0

Zhangheotherium 0001-00-0----1311001200-----01000100000013011100-0100---00--0

Maotherium 0001-00-0----1311001200-----01000100000013011100-0100---00--0

Dryolestes 0001-00-0----1301001300-----0200002000000-01110000220--000--1

Henkelotherium 00-0-00-0----1301001300-----0200002-00000-01110000220--000--1

Amphitherium --01-00-0----1301001300-----0001002000000-00110000210-0000--1

Peramus 0000-00-0----1301001300-----000100010000110011000021100000-01

1 1

Vincelestes 0000-00-0----1301001300-----0000002000000100110000210--000-01

Nanolestes 0000-00-0----1301001300-----000100210000110011000021000-00-01

Kielantherium 0000-00-0----1301101300-----000110211000110111020151100000001

1

Aegialodon -------------1-01--1300-----00011021100011-111020151100-11001

Montanalestes 0001000-0----13-2--1300-----010210211000112012021151100111002

Prokennalestes 0000020-0----1302201300-----020110111000112012021151100111002

Murtoilestes 00-----------1302201300------2021011100011-012021151100-1100-

Eomaia 0000020-0----1302201300-----020-10111000112--202-151100111--2

Juramaia 200-020-0----1302301300-----02-----110-0112-1--2-1511001----2

Kennalestes 2000100-0----1302201300-----020210211000112222031151100111002

Asioryctes 2000010-0----1302201300-----020210211000112012031151100111002

Ukhaatherium 2000020-0----1302201300-----020-10211000112012031151100111-02

Zalambdalestes 2000120-0----1302201300-----120310211000110222042051100011102

Daulestes 20---00-0----1302201300-----02021021100011-112021151100111002

Aspanlestes 00000-0-0----1302311300-----11021021100011-112012151100011002

Eoungulatum 00------0----1302421300-----11031021100011-112012151110012003

Cimolestes 2000010-0----1302311300-----020210211000112212031151100112002

Gypsonictops 2000110-0----1302311300-----120210211000112212041151100112002

Protungulatum 0000010-0----1302421300-----110210211000112112012151110012013

Erinaceus 2000100-0----1302201300-----110310211000110112022151110012112

Leptictis 2000100-0----1302221300-----110310211000112112041151100011012

Canis 0000010-1----1102301200-----0010100000000000000010210-0012100

Felis 0000010-1----1102301200-----0010100000000000000000210-0012100

Rattus 00------0----13---21300-----13-000400000000----42041100013-14

Oryctolagus 00001---0----13---01300-----13-000400000000----210410-00-3-13

Bradypus 00------0----------1500--------------0--003------------------

Tamandua --------0------------00--------------0---0-------------------

Glyptotherium 00------0----------1500--------------0--003------------------

Dasypus 00------0----------1500--------------0--003------------------

Chaetophractus 00------0----------1500--------------0--003------------------

Euphractus 00------0----------1500--------------0--003------------------

Holoclemensia 0------------1302101300-----020110211000111111021151100-21001

Sinodelphys 0--00-0-0----1302--1300-----010110011000111111-21151100121--1

Deltatheridium 0000000-0----1302101300-----000110011000111111021151100011001

2

Atokatheridium -------------1-02101300-----00011001100011-111021151100-0--01

Sulestes 0000000-0----1302101300-----000210011000111111121151100122112

Asiatherium 0000000-0----1302201300-----010310211000110111122151101122112

Kokopellia --00000-0----1302101300-----010210211000110011122151101122102

Anchistodelphys --------0----1-02101300-----02031021100011-011122051101132102

Albertatherium --------0-----302201300--------------0---1---1---------------

Didelphodon 0000000-0----1302201300-----000310211000112111122051201-32112

Pediomys 00----0-0----1302201300-----010310211000111111122051201132112

Turgidodon 0000000-0----1302101300-----010310211000111111132151201132112

Mayulestes 0000000-0----1302101300-----010310211000111111122151201131102

Pucadelphys 0000000-0----1302101300-----120310211000110111122151201132102

Andinodelphys 0000000-0----1302101300-----120310211000112111122151201032112

Didelphis 0000000-0----1302101300-----020310211000110111122151201032112

Marmosa 0000000-0----1302101300-----020310211000111111121151201032122

Caenolestes 00----0-0-----302101300-----020310211000111111132151201032122

Dasyurus 0000000-0----1302101300-----020310211000111111122151201032122

Perameles 00----0-0-----302101300-----020210211000112111132151200032122

Dromiciops 0000000-0----1302101300-----020310211000111011132051201032122

Thylacomyidae 00----0-0-----30-----00--------------00011--11132--1----3---4

Macropus 00----0-0-----3-2531300-----130300200000113301132041310043124

Acrobates 00----0-0-----3-3531300-----130210200000110101132151200042124

Phascolarctos 00----0-0-----3-3531300-----130320200000113301132041300043124

Vombatus 00----0-0-----3-3531300-----130320200000113301132041310043124

Phalanger 00----0-0-----3-3531300-----130320200000113300132151200043124

Pseudocheirus 00------0-----3-3531300-----130320200000113300132041300042124

Petauroides 00------0-----3-3531300-----130320200000113300132041300042124

Input data matrix (continued):

1111111111111111111111111111111111111111111111111111111111111

2222222333333333344444444445555555555666666666677777777778888

Taxon/Node 3456789012345678901234567890123456789012345678901234567890123

-------------------------------------------------------------------------------

Thrinaxodon 00--------------------------0---00----00-------------00000000

Massetognathus 00--------------------------033300----11-------------00000000

Probainognathus 00--------------------------0---00----00-------------00000000

Tritylodontids 00-0-0----------------------033300--0023-------------2-030000

Pachygenelus 00--------------------------0---00----00-------------30030000

Brasilitherium 00--00----------------------000000-------------------100-0000

Brasilodon 00--00----------------------000000-------------------10020000

Adelobasileus --------------------------------------00--------------0------

Sinoconodon 00-0-0---00--------0--------000000----00-0-0------00000000000

Morganucodon 00-410---00--------0--------00000100--1100-0------00000000000

1 1

Megazostrodon 00-4-0---00--------0--------00000100--1100-0------00010010000

Haldanodon 00-1-200-00--------0--------03320121--1100-0------00000010000

Castorocauda -----------------------------00-0-1---1100-0------000---100--

Docofossor 00-1020000--------10--------03320121--1100-0------00010010000

Agilodocodon 00-1020000--------10--------03320121--1100-0------00010010000

Megaconus ----------------------------033401000013-------------301400--

Cifelliodon ----------------------------0----0----1--------------31------

Millsodon ----------------------------0--3-0---013---------------------

Hahnodon ----------------------------0----0---013---------------------

Eleutherodon ----------------------------0443010-0113--------------1------

Sineleutherus ----------------------------044-010-0113--------------1------

Thomasia ----------------------------03330---0013-----------------000-

Haramiyavia ----------------------------0333010-0013-------------10010000

Arboroharamiya ----------------------------0334000-1113-------------4--400-0

Xianshou linglon ----------------------------043400--1113-------------30140000

Xianshou songae ----------------------------043400--1113----------------400--

Shenshou ----------------------------033300--0013-------------4--40000

Vilevolodon ----------------------------044400--1114-------------4--40000

Maiopatagium ----------------------------0---00---013-------------4-----00

Vintana -0--------------------------0--300---02--------------31------

Rugosodon ----------------------------033300--0023-------------20140000

Kuehneodon ----------------------------033300--0023-------------21140000

Sinobaatar ----------------------------033300--0023-------------21040000

Plagiaulacids ----------------------------033300--0023-------------21140000

Cimolodontans ----------------------------033300--0023-------------30040000

1

Hadrocodium 0--------00--------0--------00000100--1100-0------00000000000

Shuotherium 11-1-1002-0000-0--0000000-10011200----221100--0100111--------

Pseudotribos 11-1-1002-0000-0--0000000-10011200----221100--0100111--01-0--

Asfaltomylos ----0-----------------------011-0-----2211-1102--0000--------

Ambondro ----------------------------011-0-----2211-1102010000--------

Ausktribosphenos ----0-----------------------033-0-----2221-1112111000--------

Bishops ----0-----------------------033-0-----2221-1112111000--------

Teinolophos -----------------------------33-0-----2221-2113102000--------

Kryoryctes -------------------------------------------------------------

Steropodon ----------------------------033-0-----2221-2113102000--------

Obdurodon 20-1----------------0-------033300----2221-21131020005--5----

Ornithorhynchus 20-1------------------------033300---------2-1-1-2---5--5----

Tachyglossus -----------------------------------------------------5--5----

Fruitafossor 00-0-------------------------55400----0----------------02-0--

Gobiconodon 00-110---10--------0--------000100-2-11101-0------0002-030000

Repenomamus 00-110---10--------0--------000100-2--1101-0------00020030000

Spinolestes 00-110---1-1-------0--------00000-----11010-------00020020000

Amphilestes 00--------------------------000-0-----1201-0------000--------

Yanoconodon 00-0-0---10--------0--------00000120--1100-0------00030030000

Jeholodens 00-0-0---10--------0--------00000120--1100-0------00010010000

Trioracodon 00-1-0---1---------1--------00000112--1100-0------000--0300--

Priacodon 00-1-0---10--------1--------00000112--1100-0------000---300--

Tinodon 10-1-1----00---0---0----0-0002210-----2211-0------000--------

Akidolestes 20-1-1----000--0--0000000-0002220121--222100--0000000100100--

Spalacotherium 20-1-1----000--0--0000000-0002220121--222100--0000000-0------

Zhangheotherium 20-1-1----000--0--0000000-0002220121--222100--0000000200200--

Maotherium 20-1-1----000--0--0000000-0002220121--222100--0000000200200--

Dryolestes 10-001----000--0--0001000-0002220121--221100-00100000100100--

Henkelotherium 10-001----000--0--0001000-0002220121--221100-00100000--0-----

Amphitherium 111001----000--0--0001000-00022101----221111000000000---100--

Peramus 211201-0--010000--000100200001110121--221111000000000---2----

Vincelestes 20-101-0--010000--000101200001110001--221100-0000000010030000

Nanolestes 21-1-1----0-0000--101201200001110121--22111100000000010-1----

Kielantherium 20-102000-0100-0--1002011101011-0121--222111001000000--------

Aegialodon -----------------------------11---21--2221-1001000000--------

Montanalestes ----1-----------------------0---0-21--2221-1001110000--------

Prokennalestes 210212020-01001100101101000001120121--222121001110000--------

Murtoilestes 2102-2020-0-00110010110000000112-121--222121001-10000--------

Eomaia 210212020-0100110010--000-0001120121--2221210011--00000010000

Juramaia 211212020-01001100101101110101120121--222121001-1000000010-0-

Kennalestes 210212011-010011011010000-0001120121--2221210011100001002----

Asioryctes 210212011-010011001000000-0001120121--22212100111000000010000

Ukhaatherium 210212011-010011001000000-0001120121--22212100111000000010000

Zalambdalestes 20-102012-011011000000000-0001120121--222121001110000200211-0

3

Daulestes 20-112021-010011111010000-0001120121--222121001110000-00100--

Aspanlestes 20-102032-1110312100-0000-1001120121--222121001110000--------

Eoungulatum 20-102232-111031210010000-100112000---222121001110000--------

Cimolestes 210112111-010011110000000-0001120121--222121001110000---200--

Gypsonictops 210112111-010011110000000-0001120121--222121001110000--------

Protungulatum 20-102232-111031000000000-1001120001--222121001110000--------

Erinaceus 20-102122-111020--0000000-1003330001--22212111111000021030000

Leptictis 20-102111-110011110000000-1001120001--22212111111000021020000

Canis 00-001020-211011000110000-3101121001--212100-0210000020020000

Felis 00-001020-211000-00110000-3101121001--212100-02100---20020000

Rattus 00--02022-211020-----------0033100----2-------01-0---31-41111

Oryctolagus 00--02012-111030-----------01332000---2-------01-0---31-41111

Bradypus ----------------------------05540--------------------5--5----

Tamandua -----------------------------------------------------5-05----

Glyptotherium ----------------------------03330--------------------5-05----

Dasypus ----------------------------05540--------------------5-05----

Chaetophractus ----------------------------05540--------------------5-05----

Euphractus ----------------------------05540--------------------5-05----

Holoclemensia 20-2-2011-1100010010011121100112-121--222121001000000--------

Sinodelphys 211212----110------0110121100---0121--2221-100100000010010000

Deltatheridium 211202010-11000100100100102201120121--222121001000000100100--

Atokatheridium 21-2-2010-1100010010010010220112-12---222121001000000--------

Sulestes 211212010-11000100100111102201120121--222121001010000--------

Asiatherium 210212021-1111211-000100101001120121--222121001110000--0-----

Kokopellia 21-212020-1100111-100111101001120121--222121001110000---1----

Anchistodelphys 211212022-12102101010111102001120-----222111001110000--------

Albertatherium 2112-2022-2110210110011111200--2-121--222111------000--------

Didelphodon 2112-2022-22102010100201102101120121--222111001110000--0-----

Pediomys 211212022-22102110000111101001120121--222111001110000--------

Turgidodon 211212022-21102110000111102001120121--222111001110000--------

Mayulestes 211212022-22002110000101102101120121--22211100111000000010000

Pucadelphys 210212022-22112111000211111001120121--22211100111000000010000

Andinodelphys 210202022-22112111000211111001120121--22211100111000000010000

Didelphis 210202022-221120-0000211112001120121--22211100111000000010000

Marmosa 210202022-221120-0000211111001120121--22211100111000000010000

Caenolestes 210202022-221120-0010210101001120121--22211100111-00010010000

Dasyurus 211202022-221120-0010210102001120121--22211100111000010020000

Perameles 210202022-221120-0010210102001120121--22211100111-00000020000

Dromiciops 211202022-221120-0000000111001120121--22211100111000000010000

Thylacomyidae -----2042---1120-00100001--011120121--222101---11000000020000

Macropus 00-302042-211120-00100000-3001120121--222101110110---20040000

Acrobates 20-302042-211120-0010000111001120121--222101001110---00030000

Phascolarctos 20-302042-211120-00100100-3011120121--222101110110---20040000

Vombatus 20-302032-211120-00100000-3011120121--222101110110---30040101

Phalanger 20-302042-211120-00100100-3001120121--222101110110---20030000

Pseudocheirus 20-302042-211120-00100100-3011120121--222101110110---20030000

Petauroides 20-302042-211120-00100100-3001120121--222101110110---20030000

Input data matrix (continued):

1111111111111111222222222222222222222222222222222222222222222

8888889999999999000000000011111111112222222222333333333344444

Taxon/Node 4567890123456789012345678901234567890123456789012345678901234

-------------------------------------------------------------------------------

Thrinaxodon 000000----00-0-0000001-----------0000000000001000000000000-0-

Massetognathus 000000----00-0-000000100-0---0-0-00000-0---0--00000000--0-0--

Probainognathus 000000----00-0-000000------------00000-----0--01000000--0-0-0

Tritylodontids 2--2----0000-0-10--11200100000010000100000000-010000010000000

Pachygenelus 100000--0000-1-2000001-----------000000-00--0--10---0-000100-

Brasilitherium 100100----------00-00--0------------10-----------------------

Brasilodon 000000----00-0--00-00--0------------10-----------------------

Adelobasileus -----------------------------------0-------------------------

Sinoconodon 0000003311220200000001-----------100100-0---0-01------0010000

Morganucodon 0000000021000000001101-----------000100000000--11-1-000--1---

32 2

Megazostrodon 0000000011000000001001-----------20-200-00000-01101000-------

Haldanodon 0010102200000000001100-----------00020-----------------------

Castorocauda ---110-00--0-10--01100------------0020--------00010000-------

Docofossor 00101022323110000011000-----------0200--------010-0000-------

Agilodocodon 00111000020011000011000-----------020000-----1010-00000------

Megaconus 2--2--333343000-2---12-0000011101-10300-00-001011100000000---

Cifelliodon 1-0-------4----2------------2--0---030-----------------------

Millsodon ----------------------------0--0----3------------------------

Hahnodon ----------------------------2--0----3------------------------

Eleutherodon --------------0-------01111001-01-103------------------------

Sineleutherus ----------------------011110-1-01-103------------------------

Thomasia ------3323----0-3---1-1-1010--000--030-----------------------

Haramiyavia --0100-133-1--0000--1210101000000-00-0-----------------------

Arboroharamiya 2--2--334443--023---120111100-001-103---------01--10-------0-

Xianshou linglon 2--2--334443--023---120111100-001-103----11--101--100--------

Xianshou songae 2--2--334443--02----120111100-001-103----11--101--1---00---01

Shenshou 2--2--334443--023---12011-100-001-103--------10110100-0000101

Vilevolodon 2--2--3-4443--023001-201111000001-10300-0-1-0101--10000100001

Maiopatagium 2-----3--44----22--1---0-----00---10-00-0-1-01010-10000100001

Vintana 2--------24----2-----------------21020-----------------------

Rugosodon 1102--01442200103-111220100100100-002011111010-11211000000110

Kuehneodon 1102--11443200103-111220000100100-00201----------------------

Sinobaatar 2--2--024423001-0-111200000010112-0020-11-1010011211000000110

Plagiaulacids 2--2--02442300103-11120000001011200020-----0-----------------

3

Cimolodontans 2--2--12443300120-11120-0000101122002001011010011211000000110

3 2

Hadrocodium 0010003344430000001101------------0020-----------------------

Shuotherium --------3-----0-------------------002------------------------

Pseudotribos 100-0-003310000--01100------------0020-------101--00--0010001

Asfaltomylos --------------0-------------------0-2------------------------

Ambondro --------------0-------------------0-2------------------------

Ausktribosphenos --------3----00-------------------0-2------------------------

Bishops -------03--0-00-------------------002------------------------

Teinolophos ----------------------------------0-2------------------------

Kryoryctes -------------------------------------------------------------

Steropodon --------3--3----------------------0-2------------------------

Obdurodon 2--2--333443--0----1--------------0020-----------------------

Ornithorhynchus 2--2--334443--0----1-------------0----11110111110100000010001

Tachyglossus 2--2--334443--------------------------11110111110100000010001

Fruitafossor 100100223332000-001100-----------30100--------01000010-------

Gobiconodon 1001001122000000001010-----------20021-0-1--01010100110000-10

Repenomamus 1001001122000000001010------------002100110101010100110000110

Spinolestes 1001012122320000000010------------0021---11101010-00110-00110

Amphilestes ---00--21--0000------0------------002------------------------

Yanoconodon 0000013333430001001110------------002000-10001010100000000110

Jeholodens 10010-3322320000001111------------002000100-01010200000000110

Trioracodon 000000123321000--01100------------0020-----------------------

Priacodon 0-0000122221000--01101-----------10020-----------------0-----

Tinodon ---1---22--1--0-------------------0-2------------------------

Akidolestes 1001100001000000001110------------0020-----0-0011-00000100110

Spalacotherium ---100-000-0000000--10------------0020-----------2-----------

Zhangheotherium 1001002200000100001110-----------20020-1110-00011211000100110

Maotherium 1001012200000100001110------------0020-11-0000011-11000-00110

Dryolestes 0000001100000000301110-----------20020-----------------------

Henkelotherium 00-0-0110000000--0---0------------0-20-----0--011-1100--0---0

Amphitherium ---0-0-000-0-00-------------------0-2------------------------

Peramus -00000003310-00--0---0------------0-20-----------------------

Vincelestes 001000333342000030--00------------0-2001111010011-110-1201-10

Nanolestes --0010-02--0-00--0---0------------002------------------------

Kielantherium ---0---12--0--0------0------------002------------------------

Aegialodon ----------------------------------002------------------------

Montanalestes -------03--0-00-------------------0-2------------------------

Prokennalestes -0101-0033-0000-00-1-0------------0020-----------------------

Murtoilestes -0--------------------------------002------------------------

Eomaia 000000003310010000-100------------0020111-1010011211001200-10

Juramaia 001000003310000000-100------------00201-11101001--1--0-------

Kennalestes 0010101033200100001101------------0020011--------------------

1

Asioryctes 001010113321010000-101------------00200111101--11-110--------

Ukhaatherium 000001113321010030-101------------0020-11---1-011-1100--0---0

Zalambdalestes 001100113321010200-111------------0020---11010011-1100--0---0

22 3

Daulestes 0001001133200-0--0-100------------0020-----------------------

Aspanlestes -----0-033-0--0----1-0------------002------------------------

Eoungulatum -------1332---0----1-0------------002------------------------

Cimolestes 0-000-113321-00----1-0-----------20020-----------------------

Gypsonictops 0-000-113320-00----1-0-----------20020-----------------------

Protungulatum 0-0000113321-00----100-----------20020-----------------------

Erinaceus 1001013333420002001110-----------2002011111010011211001200-11

Leptictis 000101113321000200-100------------0020--------011-110-1200-10

Canis 0000002332310000000100-----------201201111101101121100120--10

Felis 0000013443430-00000100-----------201201111101101121100120--10

Rattus 2--2--333343---1-02112-----------2002011111010011211001200-10

Oryctolagus 2--2--333343--0--02112-----------2113011111010011211001201-10

Bradypus 1--2------00------01-------------301-01111111101121110120--10

Tamandua 1--2--------------01-------------3---01111111101121110120--10

Glyptotherium 10-2------00------01-------------3010011111-1101121110120--10

Dasypus 10-2------43------01-------------301001111111101121110120--10

Chaetophractus 10-2------43------01-------------301001111111101121110120--10

Euphractus 10-2------43------01-------------301001111111101121110120--10

Holoclemensia --------------0-------------------002------------------------

Sinodelphys 000000113211120-10-100------------0020-------001121-00120--10

Deltatheridium 0000002222211100-12100-----------20020-----------------------

Atokatheridium ----------------------------------002------------------------

Sulestes ----0-222221-10----1--------------002------------------------

Asiatherium 000-0-222221010-----0-------------0020------1001121100--0----

Kokopellia 0-000--222-1-10--0-100-----------2002------------------------

Anchistodelphys ----------------------------------002------------------------

Albertatherium ---------2------------------------002------------------------

Didelphodon -0-000222221-00----1-0-----------20020-----------------------

Pediomys ---00-222221100------0-----------20020-----------------------

Turgidodon ---000222221-00---21-0-----------2002------------------------

Mayulestes 000000222221100111-100------------002011111-10011-11001201-10

Pucadelphys 0000002222211101112100------------002011111110011211001201-10

Andinodelphys 000000222221110111-100------------0020--------0---------0---0

Didelphis 0000002222211101112100-----------2002011111110011-11001201-10

Marmosa 0001002222210001112100------------002011111110011211001201-10

Caenolestes 0000012222210100202100--------0---002011111-10011211001201-10

Dasyurus 0000013322311000112100------------002011111110011211001201-10

Perameles 0000002222210100212101------------002011111110011211001201-10

Dromiciops 0001012222210100212100------------002011111-10011211001201-10

Thylacomyidae 00---1222221--0-2-210-------------002011111-10011211001201-10

Macropus 1-----332232-110202112----1-1-0---002011111110011211001201-10

Acrobates 00010-2222310000202110------------002011111-10011211001201-10

Phascolarctos 10----332233-200102111------------002011111110011211001201-10

Vombatus 20----332233-201-02112------------002011111110011211001201-10

Phalanger 00010-2222220210202111------------002011111110011211001201-10

Pseudocheirus 10010-3322320000202111------------002011111110011211001201-10

Petauroides 00010-3322320000202111------------002011111110011211001201-10

Input data matrix (continued):

2222222222222222222222222222222222222222222222222222222333333

4444455555555556666666666777777777788888888889999999999000000

Taxon/Node 5678901234567890123456789012345678901234567890123456789012345

-------------------------------------------------------------------------------

Thrinaxodon 000000-0000000000000000000000-----000000--0000000000000000000

Massetognathus ----0-----00----0000000000000-----1000-0--00000---0--0---0-00

Probainognathus ----0-----00----------0----0------10---0--000-0---0--0---0-00

Tritylodontids 00000000000000000000001000000-----2000010-0000000000000000000

1

Pachygenelus 00-000-00000000000000010000001000-210001----00-0000000-----00

Brasilitherium -------------------------------------------------------------

Brasilodon -----------------------0---0---------------------------------

Adelobasileus --------------------------------------------0----------------

Sinoconodon 00000000000000000000001000000------------------000-00-0---00-

Morganucodon 00-100-00000-0000000001000000-----2100010---000010000010-0000

Megazostrodon 0--100000000-000000000100000--0--02100010-000000100000-000000

Haldanodon ---100101000-00-0000012000010-----21-0-------000-00000-0-0-0-

Castorocauda -----------------00001200001000000210000000000000000001011000

Docofossor -----------------------0000100101021000-0-0000-00000001011001

Agilodocodon 1101001----0---0-00-0100011110001021000000000000-000001011000

Megaconus 11-0-0------00-00000-00001000----021-0--0000---0-00000-200210

Cifelliodon -------------------------------------------------------------

Millsodon -------------------------------------------------------------

Hahnodon -------------------------------------------------------------

Eleutherodon -------------------------------------------------------------

Sineleutherus -------------------------------------------------------------

Thomasia -------------------------------------------------------------

Haramiyavia -------------------------------------------------------------

Arboroharamiya 01-------------------------100---120--0----00--0-00---100--00

1

Xianshou linglon -----------------000110000-10010012100201-0000---0000--00-000

Xianshou songae -1-----------0---0001100001100--012100201--0-000---000100-000

Shenshou 11000000011000000000110000110010012--0201-00-000-00000100-000

Vilevolodon 11000000011000000000110000110010012100201-000000-000001000000

Maiopatagium 11000000011000000000110000110010012100201-000000-000001000000

Vintana -------------------------------------------------------------

Rugosodon 11011001111001100100-000010110001021-011000000-1-001002011001

Kuehneodon -------------------------------------------------------------

Sinobaatar 1101100111100110010020000101100010210011000000011101002011001

1

Plagiaulacids ---------------------------------0----------------1----------

Cimolodontans 1101100111100110010010000001100--02101110000000111-1002011001

2 1

Hadrocodium ---------------------------0---------------------------------

Shuotherium -------------------------------------------------------------

Pseudotribos ------------0----0---12000000-----211---00000-10-0000000-00--

Asfaltomylos -------------------------------------------------------------

Ambondro -------------------------------------------------------------

Ausktribosphenos -------------------------------------------------------------

Bishops -------------------------------------------------------------

Teinolophos -------------------------------------------------------------

Kryoryctes ------------------000121000----------------------------------

2

Steropodon -------------------------------------------------------------

Obdurodon ----------------------------------21---1----11---------------

Ornithorhynchus 000000101000000000000120000001001-211111000011100000022011002

Tachyglossus 000000101000000000000120000001001-211111010011100000022011002

Fruitafossor 0--000011000-00100000120001001001021------0000---00--0100-100

Gobiconodon 11121111111001110000011100010000--21002100001000000002110-000

Repenomamus 111211111110011100000111000100----210021000010000000021100000

Spinolestes 11121111111001110100011100010----021002100001001-111111100000

Amphilestes --------------------------------------------------0----------

Yanoconodon 111211111110011100000101010000001-210021000000000000001100000

Jeholodens 111211111110011100--01010100000010210021000000000000001100000

Trioracodon -------------------------------------------------------------

Priacodon -------------------------------------------------------------

Tinodon -------------------------------------------------------------

Akidolestes 110211111110011101111101110100001-21111201000011-000002100102

Spalacotherium --------------------------------------------------1------0---

Zhangheotherium 110211111110011101111101110100001021112200000001-111002100100

Maotherium 110211-11110011101111101110100001021112200000001-1-100210-100

1

Dryolestes -------------------------------------------------------------

Henkelotherium 11021111-110-111011-1001110210001-21-022000000011100002110000

Amphitherium -------------------------------------------------------------

Peramus -------------------------------------------------------------

Vincelestes 1-0211011110011101010001110-00001-210122000000011100002110000

Nanolestes -------------------------------------------------------------

Kielantherium -------------------------------------------------------------

Aegialodon -------------------------------------------------------------

Montanalestes -------------------------------------------------------------

Prokennalestes -------------------------------------------------------------

Murtoilestes -------------------------------------------------------------

Eomaia 110221011110021101112002210210001021112200000001-101102111000

Juramaia 1102210--110-21-01----02210200---0---------------------------

Kennalestes -------------------------------------------------------------

Asioryctes ----------------0-----0---0-11000------2-0--00-------0--1-000

Ukhaatherium -10221011110-211011120022102100-1-210-2200000001-101102111000

Zalambdalestes 1-0221011110-21101112002210210001021112200000001-101102111210

Daulestes -------------------------------------------------------------

Aspanlestes -------------------------------------------------------------

Eoungulatum -------------------------------------------------------------

Cimolestes -------------------------------------------------------------

Gypsonictops -------------------------------------------------------------

Protungulatum -------------------------------------------------------------

Erinaceus 11021101111012110111200221021000102111221-0000011101112101210

2

Leptictis 1--22--111-012110111200221021----02111221-0000011101112211210

Canis 11021201111012111111200221121000002111221-0000011001101111110

Felis 11021201111012111111200221121000002111221-0000011001101111110

Rattus 11022101110001101000210221120000102111211-0010111101112111100

Oryctolagus 110221011100020010112002210200001-2111201-0010111101112101110

Bradypus 110212011121021111112002211210000-2111321-1100011001101201100

Tamandua 110212011121021111012002211210000-2111321-1100011001101101100

Glyptotherium 110212011120021111012002211210000-2111321-1100011001101201210

Dasypus 110212011120021111012002211210000-2111321-1100011001111101210

Chaetophractus 110212011120021111012002211210000-2111321-1100011001111101210

Euphractus 110212011120021111012002211210000-2111321-1100011001111101210

Holoclemensia -------------------------------------------------------------

Sinodelphys 11-23101111012-111112-02211211110021----00000001-10--0-100000

Deltatheridium -------------------------------------------------------------

Atokatheridium -------------------------------------------------------------

Sulestes -------------------------------------------------------------

Asiatherium 11-231011110-21101112002211211110021012200000001-101101100100

Kokopellia ---------------------------------0---------------------------

Anchistodelphys -------------------------------------------------------------

Albertatherium -------------------------------------------------------------

Didelphodon -------------------------------------------------------------

Pediomys -------------------------------------------------------------

Turgidodon -------------------------------------------------------------

Mayulestes 11-23101111012111111200221121-1---211122000000011101101110000

Pucadelphys 11-2310111101211111121022112------211122000000011101101110000

Andinodelphys -----------------------------------------------------0-------

Didelphis 1102310111101211111120022112121100211122000000011101101110000

1

Marmosa 1102310111101211111120022112121100211122000000011101101110000

Caenolestes 1102310111101211111120022112121100211122000000011101101110000

Dasyurus 1102310111101211111120022112121100211122000000011101101110000

Perameles 1102310111101211111120022112121100211122000000011101101100100

Dromiciops 1102310111101211111120022112121100211122000000011101101110000

Thylacomyidae 1102310111101211-11120022112111100211122000000011101101110000

Macropus 1102310111101211111120022112111100211122001000011101101210010

Acrobates 110231011110121111112002211211110-211122000000011101101110000

Phascolarctos 110231011110121111112002211211110-211122001000011101101110000

Vombatus 1102310111101211111120022112111100211122001000011101101100100

Phalanger 1102310111101211111120022112111100211122000000011101101110000

Pseudocheirus 1102310111101211111120022112111101211122000000011101101110000

Petauroides 1102310111101211111120022112111100211122000000011101101110000

Input data matrix (continued):

3333333333333333333333333333333333333333333333333333333333333

0000111111111122222222223333333333444444444455555555556666666

Taxon/Node 6789012345678901234567890123456789012345678901234567890123456

-------------------------------------------------------------------------------

Thrinaxodon -0000-0000000000000000-000000000110000000---000000000-00-----

Massetognathus --------------------------------------------0---0--00-00-----

Probainognathus ------------------------------------------------0--00-00-----

Tritylodontids -0000-00-000000000000000000000001--000-0-00-000000000-00-0---

Pachygenelus ------------------------------------------------0000000100-0-

Brasilitherium -------------------------------------------------0-00-0--0---

Brasilodon -------------------------------------------------0-00-0--0---

Adelobasileus -------------------------------------------------00000-------

Sinoconodon ------------------------------------------------000000010000-

Morganucodon --000-00000000000000000000000000---0--------0--0000001010000-

11

Megazostrodon -0000-00-0000-00000-00--0---0-0-1-000000000-000000-0010100---

Haldanodon --------------------------------------------0---000001020011-

Castorocauda -0100-01-1101100000000000000--00-0--0-00000-01100---010------

Docofossor 10100-0101101100000000000000000010000000100001001----1-------

Agilodocodon -0100-0101101100000000000000000010000001100001001----1020011-

Megaconus -00-0--0000-000000000---00-0--0---00-0------00100----1-------

Cifelliodon -------------------------------------------------0000101-111-

Millsodon -------------------------------------------------------------

Hahnodon -------------------------------------------------------------

Eleutherodon -------------------------------------------------------------

Sineleutherus -------------------------------------------------------------

Thomasia -------------------------------------------------------------

Haramiyavia -------------------------------------------------------------

Arboroharamiya -00-------------0-------------------0----001-0-01------------

Xianshou linglon -00------00-0-00010000-0000-000000000000100100-01------------

Xianshou songae -000-----00-0-00010000-0000-000000000000100100101------------

Shenshou -000-0---00-0-00-10000-0000-0000000000001001001011--0101-110-

Vilevolodon -000-00000000-00010000-00000000000000000100100-0-1-00101--10-

Maiopatagium -000-000000-0-000---00-00---00000-000-00---100-0----0--------

Vintana -------------------------------------------------00000010111-

Rugosodon 0001210010001000012001000111000101100011100002101-0----------

Kuehneodon -------------------------------------------------------------

Sinobaatar 000121001000100001200100011100010110001110000210100001010110-

Plagiaulacids -------------------------------------------0-----------------

Cimolodontans 000121001000202111200100111100110110001110000210100001010010-

1

Hadrocodium -------------------------------------------------10000020101-

Shuotherium -------------------------------------------------------------

Pseudotribos --------------------------------------------0---0------------

Asfaltomylos -------------------------------------------------------------

Ambondro -------------------------------------------------------------

Ausktribosphenos -------------------------------------------------------------

Bishops -------------------------------------------------------------

Teinolophos -------------------------------------------------------------

Kryoryctes -------------------------------------------------------------

Steropodon -------------------------------------------------------------

Obdurodon ------------------------------------------------100002110110-

Ornithorhynchus 10000-0000000010110000000001000110000110100-1100100002110010-

1

Tachyglossus 10000-0000000010110000000001000110000110100-1100100002110010-

1

Fruitafossor -0-0--------0000000000000-0-0000-----------001--0----10---11-

Gobiconodon -0000-00-000010001000000000000000--------0--0010000001120111-

Repenomamus -0000-000000010001000000000000000-0-0--1-00-0110000001120111-

Spinolestes 00000-0000001100010000000000--0------0-1----010-10---1120111-

Amphilestes -------------------------------------------------------------

Yanoconodon -0000-000000010001000000000000000-0-0-01--0-0200000001020-11-

Jeholodens -0000-0000000-000100000000000000000000001000000000000102-011-

Trioracodon ------------------------------------------------0------------

Priacodon ------------------------------------------------0-----0----1-

Tinodon -------------------------------------------------------------

Akidolestes 1001000000001-00201001000111000010000121000-021010-001020--1-

Spalacotherium -------------------------------------------------------------

Zhangheotherium -001000000001030201001000111000010000020000002101----1020111-

Maotherium -0010000000-103020100----1110000100000200000021010-001020111-

1

Dryolestes ------------------------------------------------1------------

Henkelotherium -10---------1--------------------------0----0-1011---1020-11-

Amphitherium -------------------------------------------------------------

Peramus -------------------------------------------------------------

Vincelestes -1020-00-10010302010110001110100--0---210---0-00111002020011-

Nanolestes -------------------------------------------------------------

Kielantherium -------------------------------------------------------------

Aegialodon -------------------------------------------------------------

Montanalestes -------------------------------------------------------------

Prokennalestes -------------------------------------------------------------

Murtoilestes -------------------------------------------------------------

Eomaia -01221000212203020101301111101000001002100001200111-0-1----2-

Juramaia -------------------------------------------0-----------------

Kennalestes ----------------2--------------------------------110021221122

Asioryctes -01221000212203020101301111101000000-020000-1-001110021221122

Ukhaatherium -0122100021220302010130111110100000---200---1--01--00-1-2--22

Zalambdalestes --1221000212203-201013-0113100000000002000001-001110031221122

Daulestes -------------------------------------------------110021201120

Aspanlestes -------------------------------------------------------------

Eoungulatum -------------------------------------------------------------

Cimolestes -------------------------------------------------------------

Gypsonictops -------------------------------------------------------------

Protungulatum --222003-21220312-10130111312011-----------------------------

Erinaceus -122200302122051201013011131201100000020010012001110031211120

Leptictis -1222003-2122-31201013011131201100---0200-001--01110031211120

Canis -2221202021220510010130111--30110-0-0020000012001110030211131

Felis -2221202021220510010130111--30110-0-0020000012001110030211131

Rattus -122100302122050001011011-31101101010001000012001110020101100

Oryctolagus -0-2111302122050001013010131101101010001000-12001110020101100

Bradypus -1221201021220510010130111--30110-0-0020000-12001110030211131

Tamandua -2221201021220510010130--1--30110-0-0020000-12001110031211131

Glyptotherium -2221201021220510010130111--30110-0-0020000-12001111030211131

Dasypus -2221202021220510010130111--30110-0-0020000-12001111030211131

Chaetophractus -2221202021220510010130111--30110-0-0020000-12001111130211131

Euphractus -2221202021220510010130111--30110-0-0020000-12001111130211131

Holoclemensia -------------------------------------------------------------

Sinodelphys -01212010--12042202011021-211000110110210000020011--0--------

Deltatheridium --12--------2-------110212-1--00----------------11100-020112-

Atokatheridium -------------------------------------------------------------

Sulestes -------------------------------------------------------------

Asiatherium -01---------2------------------------------00-001110-30201120

Kokopellia -------------------------------------------0-----------------

Anchistodelphys -------------------------------------------------------------

Albertatherium -------------------------------------------------------------

Didelphodon ---21201-10120311010110212211011------------------10-30201120

Pediomys ---21201-10120422020110212211011-----------------------------

Turgidodon -----------------------2-------------------------------------

Mayulestes -012--------20422020110212111011------210---0-001110030201120

Pucadelphys -0121301010120422020110212211011-101-0210---0-001110030201120

Andinodelphys ------------2-------------------------------0---111003-201120

Didelphis 0012130101012042202111021231201111011021001002001110030201120

Marmosa 0012130101012042202111021221201111011021001002001110030201120

Caenolestes -01213020-012042201011021221101111000021010002001110030201120

Dasyurus -0121312010120510010121213-1101111000021010002001110030201120

Perameles -01213020-1120510020121213-1101111010020010002011110030201120

Dromiciops -01213020-1120510020121213-1201111010021001002001110030201120

Thylacomyidae -012130-0---20510020121213-1--11--0-002-0-00020111100302-112-

Macropus -01213120-1120510010121213-1101111010020000002011110030211131

Acrobates -01213020-1120510020121213-12-111101002-0-1-02011110030211131

Phascolarctos -01213020-1120510020121213-1101111010021001-02011210030211100

Vombatus -01213020-1120510020121213-110111101002100100201121003021110-

Phalanger -01213020-1120510020121213-1201111010021001002011210030211131

Pseudocheirus -01213020-1120510020121213-1201111010021001002011210030211131

Petauroides -01213120-1120510020121213-1201111010021001002011210030211131

Input data matrix (continued):

3333333333333333333333333333333334444444444444444444444444444

6667777777777888888888899999999990000000000111111111122222222

Taxon/Node 7890123456789012345678901234567890123456789012345678901234567

-------------------------------------------------------------------------------

Thrinaxodon ---0000--000--00-0-00-000-0000010-1000000---0----0-0000-00--0

Massetognathus ---1000--000--00-0-00-000-000001001000000---0----0-0000-00--0

Probainognathus 0--1000--000--00-0-00-000-000001001100000---0----0-0000-00--0

Tritylodontids 0-01111--1000000-00000000-000001000000100---000000-0000000-01

Pachygenelus 0-01111--1-00000-0000-000-000001011000000---000000-0000010000

Brasilitherium -----1--------------------101-------00-----------------------

Brasilodon -----1---------------------0--------00------------------1-001

Adelobasileus --0221100-----0000001-11--000000010010000----0000000000010000

Sinoconodon 000221100100000000001011101010000000001000-000000000000010001

Morganucodon 000231111201000000001011101010001101001110-010000000000010001

Megazostrodon 0002311012010-00000010111010000011-1001110--10000000000010001

Haldanodon 00023110030100000000101210000-001102101110-010000000000010011

Castorocauda -------------------------------------------------------------

Docofossor -------------------------------------------------------------

Agilodocodon -0-231100-----0000001-12--------1--2101110-0--0000000---10011

Megaconus -------------------------------------------------------------

Cifelliodon 00-231100----------01-11-0000--01---11------1000000--0001-0-1

Millsodon -------------------------------------------------------------

Hahnodon -------------------------------------------------------------

Eleutherodon -------------------------------------------------------------

Sineleutherus -------------------------------------------------------------

Thomasia -------------------------------------------------------------

Haramiyavia -------------------------------------------------------------

Arboroharamiya -------------------------------------------------------------

Xianshou linglon -------------------------------------------------------------

Xianshou songae -------------------------------------------------------------

Shenshou -0-231-01-------------1---------------------------------100--

Vilevolodon -002-1-01-----------------------------------------------10---

Maiopatagium -------------------------------------------------------------

Vintana 0002311113111111010010121-000--011121-1111--1000000-010110--1

Rugosodon -------------------------------------------------------------

Kuehneodon -------------------------------------------------------------

Sinobaatar 000----------------------------------------------------------

Plagiaulacids ---231111301--000000101211101100211---11110-10-00000000010001

Cimolodontans 0002311113010010000010121110110021121011100010000000000010001

2 1 1 1 11 1

Hadrocodium 000231112301000000001-111010-100110210101100100000000000100-1

Shuotherium -------------------------------------------------------------

Pseudotribos -------------------------------------------------------------

Asfaltomylos -------------------------------------------------------------

Ambondro -------------------------------------------------------------

Ausktribosphenos -------------------------------------------------------------

Bishops -------------------------------------------------------------

Teinolophos -------------------------------------------------------------

Kryoryctes -------------------------------------------------------------

Steropodon -------------------------------------------------------------

Obdurodon 000231111-----0000001-111-1101001112101110--10000000000000-00

Ornithorhynchus 000231111411000000001011101101001112101110-110000000000000-00

Tachyglossus 000231111411000000001011101101001112101110-110000000000010-00

Fruitafossor -------------------------------------------------------------

Gobiconodon 000231111301000000001-1211200000111100111110200000011000111-1

Repenomamus 000231111301000000001-121120000011110011111020000-01100011111

Spinolestes 000231111-----0000001-12-1------11-11111-11020000-01100011111

Amphilestes -------------------------------------------------------------

Yanoconodon -00--------------------------0-------------------------------

Jeholodens 0002311-130100--0---10--1--------------------0--0-0----------

Trioracodon --02311---------0--0-0--1010---0----------------0--0-0---1--1

Priacodon -00231111301--000000101-12100--0111100--1000--000---00--11001

Tinodon -------------------------------------------------------------

Akidolestes -------------------------------------------------------------

Spalacotherium -------------------------------------------------------------

Zhangheotherium 0012311112----1101001-1-1-10---0--12101111002000000000001-0-1

Maotherium 0012311112----1101001-1---10-100-11000111---2000000000001---1

Dryolestes -------------------------------------------------------------

Henkelotherium 00-23111-3-----------------------------------0---------------

Amphitherium -------------------------------------------------------------

Peramus -------------------------------------------------------------

Vincelestes 00123-112400-111010010111010010011021111110020110000000011002

1

Nanolestes -------------------------------------------------------------

Kielantherium -------------------------------------------------------------

Aegialodon -------------------------------------------------------------

Montanalestes -------------------------------------------------------------

Prokennalestes ---23111251111110100102-10-----13-1-111011----11001-----110-2

2

Murtoilestes -------------------------------------------------------------

Eomaia -0----1--51-11----------1-------------------------------11---

Juramaia -------------------------------------------------------------

Kennalestes 102231112511--21021100231-2001013-----10110020100010010011002

4

Asioryctes 1022311125-1--2102110-241-2001013---1110110020100010010011002

Ukhaatherium 1---3---------2--21----4-0----0-------1--------0-0---1------2

Zalambdalestes 1022311125-1--2102110024112001013---1110110020100010010011002

Daulestes 102231112511---10-----2-----01013-------110-201100--0-001----

Aspanlestes ----3---------1-0-0-1----0-----1---------------00-1----------

Eoungulatum ----3---------1---0-1----0--------------------000------------

Cimolestes ----------------------------------------------0--------------

Gypsonictops -------------------------------------------------------------

Protungulatum ---2311125-1--1-0-0-00--00-----1--------------11001------1--2

1

Erinaceus 102231112511112102110023113001113---1110110020110010010011002

Leptictis 102231112511111102010023113001113---1110110020110010010011002

Canis 1022311125111-11-3--0-23111101113-----101100200001000000110-1

Felis 1022311125111-11-3--0-23111101113-----101100200001000000110-1

Rattus 2012311125111111010000241101011131--2-10110010100110010021111

Oryctolagus 20-2311125111111010000241101011131--2-10110010000110010011101

Bradypus 1022311125111-11-3--0-23021101013-----101100200000000000110-1

1

Tamandua 1022311125111-11-3--0-231-1101013-----101100200100000000110-1

Glyptotherium 1022311125111-11-3--0-230-1101013-----10110020000-000000110-1

Dasypus 1022311125111-11-3--0-23011101013-----101100200000000000110-1

2

Chaetophractus 1022311125111-11-3--0-23021101013-----101100200000000000110-1

1

Euphractus 1022311125111-11-3--0-23021101013-----101100200000000000110-1

1

Holoclemensia -------------------------------------------------------------

Sinodelphys -------------------------------------------------------------

Deltatheridium ---231112510--110100212312---1013---2-10110-2-00001-----11011

Atokatheridium -------------------------------------------------------------

Sulestes ---231112510--1-0----12-1---------------------000--------1-1-

Asiatherium 10-2311125----1111-0--23--4101013-----10-10-2--0-01-001111---

Kokopellia -------------------------------------------------------------

Anchistodelphys -------------------------------------------------------------

Albertatherium -------------------------------------------------------------

Didelphodon 102231112--0----1--0-1--11-----1------1------0-00-1-------011

Pediomys --123111---0----0--0-1---1-----1------1--------00-1-------011

Turgidodon --223111---0----0--0-1--11-----1------1--------00-1-------0-1

Mayulestes 1112311125----110100--24110101013-----10110021001010000111111

Pucadelphys 111231112511--1101002124110101013-----10110021001010000111111

Andinodelphys 1122311125----1101002-24110101013-----10110021001010000011111

Didelphis 111231112511111111002123110101013-----10110020000010001111011

1

Marmosa 111231112511111111002123111101013-----10110020000010001111011

Caenolestes -11231112511111113002-23110101013-----10-10020000000002111011

Dasyurus 113231112511111113002123101101013-----10110020000010002111011

Perameles 211231112511111113002-23114101013-----10110020000010002111011

Dromiciops 111231112511111113002-231-4101013-----10110020000010002111001

Thylacomyidae 113231112511111113002-2310-101013-----1011002-000010002-1---1

Macropus 303231112511111113002-23114101013-----10110020000010003111001

Acrobates 303231112511111113002-23114101013-----10110020000010001111011

Phascolarctos 103231112511111113002-23114101013-----10110020000010002111011

Vombatus 003231112511111113002-23023101013-----10110020000010001111001

Phalanger 303231112511111113002-23114101013-----10110020000010003111001

Pseudocheirus 303231112511111113002-23114101013-----10110020000010003111001

Petauroides 303231112511111113002-23114101013-----10110020000010003111001

Input data matrix (continued):

4444444444444444444444444444444444444444444444444444444444444

2233333333334444444444555555555566666666667777777777888888888

Taxon/Node 8901234567890123456789012345678901234567890123456789012345678

-------------------------------------------------------------------------------

Thrinaxodon -0-0000000000000000000000000000000000000000000000000000-00000

Massetognathus -0-1010000000000000100000000000030000000000000001000000-00000

Probainognathus -0-001000000-000000100000000000000000000000000001000000-00000

Tritylodontids 00-0021000000000000100000000001030100000000000002001000-10000

1 1

Pachygenelus -1-0021001100000000100000000100000101010100000001000000-10000

Brasilitherium -------------------------100100--0101010100000001000-00--0000

Brasilodon 010----------------------100100--01010-0100-000010-0-00-20000

Adelobasileus -10---------0-----------0--0-0-000---------00--0200-------000

Sinoconodon 0101021001100000000100000100101000101000100000002000100020000

Morganucodon 0100021002100000000100000100101000101010100000002000100020000

Megazostrodon 01-00210-21---0--001-000010-1--000101010100000--2-0010-020000

Haldanodon 01000----21000---00--00002011-1000101010100000002000100020100

Castorocauda --------------1110010000---------0------10--00--2----0--2----

Docofossor -----------------------------------------00------------------

Agilodocodon 01000210-21---1110010000-2--1---0-------100----------0002--0-

Megaconus ---00-----1---011--10000------------------0------------------

Cifelliodon 010----------------------00000--3011300010-001012--0-0-221--0

Millsodon -------------------------------------------------------------

Hahnodon -------------------------------------------------------------

Eleutherodon -------------------------------------------------------------

Sineleutherus -------------------------------------------------------------

Thomasia -------------------------------------------------------------

Haramiyavia -----------------------------------------00-------------2----

Arboroharamiya -------------------------------------------------------------

Xianshou linglon ----------1------------------------13---101---0--------------

Xianshou songae ----------1--------------------------------------------------

Shenshou ----------1--------------20------0------101-010-------0-----0

Vilevolodon ---00---0-----1110010000----0----00-3010101-011------0012---0

Maiopatagium -------------------------20-012--0113010-01-011------00-2----

Vintana -11----------------------00121---01110000000011-2120-00220--0

Rugosodon -----------1------------------------------0------------------

Kuehneodon -------------------------------------------------------------

Sinobaatar ----------1--------2---00-----------------0------------------

Plagiaulacids 111------21-0----------0-00-201001102000100-0000202-112220200

Cimolodontans 11032312121100111--211100000101001102000100200002021112220200

12 1

Hadrocodium 010------21-0------1----02012-1030101000100000012-2-10-020101

Shuotherium -------------------------------------------------------------

Pseudotribos -----------------------------------------0--------------2---1

Asfaltomylos -------------------------------------------------------------

Ambondro -------------------------------------------------------------

Ausktribosphenos -------------------------------------------------------------

Bishops -------------------------------------------------------------

Teinolophos -------------------------------------------------------------

Kryoryctes -------------------------------------------------------------

Steropodon -------------------------------------------------------------

Obdurodon -0--------1-0-----------03012-10001-40-110021121202011222-001

Ornithorhynchus -0-3231212110011101210100301101000124001100211212020112221001

Tachyglossus -0-32312121100111012101003012110201240011-0211212021112221001

Fruitafossor -----------------------------------------00------------2-1---

Gobiconodon 011-------1-1----12----00200211000111000001000112001111221210

Repenomamus 01-------21-1----12-----0200211000111000001000112001111221210

Spinolestes 011------21-1----12------2--2---0-------001-----2-----122----

Amphilestes -----------------------------------------1-------------------

Yanoconodon ---32312121---11012210100-00-----0------110-----2----1022----

Jeholodens -------21--------1221------------01-10--110----0-----1022---0

Trioracodon 0-----------0-----------0-------0--------1-----------------0-

Priacodon 01-------2--0-----------020-----001------10-------1--1022-10-

2

Tinodon -------------------------------------------------------------

Akidolestes ---------------------------------0------100-0---2------12----

Spalacotherium -----------------------------------------0-------------------

Zhangheotherium 1-1------21------1------0------0--1-----100-------2----12--2-

Maotherium 1----------------12-----0201--20-0--22--100000----2----12-02-

Dryolestes ----------------------------------1---10-0-------------------

Henkelotherium ------------------------------1-----20001-0--1----------20---

Amphitherium -------------------------------------------------------------

Peramus -------------------------------------------------------------

Vincelestes 111------21-0-----------0101101000102010000001012000100020200

Nanolestes -------------------------------------------------------------

Kielantherium -------------------------------------------------------------

Aegialodon -------------------------------------------------------------

Montanalestes -------------------------------------------------------------

Prokennalestes 1--------2--1----------------------------------1-----------1-

Murtoilestes -------------------------------------------------------------

Eomaia ------------------------0--------02--0--100-0-0-2---10--20---

Juramaia ------------------------------------------0------------------

Kennalestes 110------21-2------321300-0110200020200--00-01012121100020220

Asioryctes 110------21-2------32130020110200020200-1001010-2121100020220

Ukhaatherium 1-1---------2-------21--0-0-----0--------00-010---21---0-----

Zalambdalestes 111------21-2------32130020110200020201-100101012111100020210

1

Daulestes ----1----2--1------3---00-01-1--002-100-100-000-212110-020---

1

Aspanlestes 1-----------1---------------------2--------------------------

Eoungulatum 1-----------1------------------------------------------------

Cimolestes -------------------------------------------------------------

Gypsonictops -------------------------------------------------------------

Protungulatum 1-----------2----------------------------------------------1-

Erinaceus 11121311121220111014213002111130002020011001101121211-0221210

Leptictis 110------21-20-----321301211112000203000100110112121110020210

Canis 1202131112122011101421311201114010213000100110112110100220221

1

Felis 1202131112122011101421311201114010213000100110112110100220221

Rattus 1102131112122011101421300201112120214001100110112120110221321

Oryctolagus 11021311121220111015213000011111212030011-0211112120110221321

Bradypus 12021311121220111014213113011110302101001-0110012110110220-21

Tamandua 12021311121220111014213113011110302101002-0110212110110220-21

Glyptotherium 120----11--22011101421311301111030210100100110012121110220-21

Dasypus 1202131112122011101421311301111130210100200110212121110220-21

Chaetophractus 1202131112122011101421311301111130210100200110212121110220-21

1

Euphractus 1202131112122011101421311301111130210100200110212121110220-21

1

Holoclemensia -------------------------------------------------------------

Sinodelphys ---------------------------------0------10--0---2----0--2-3--

Deltatheridium 11-------2--1-----------02-11-2-002-2010100-000121---0-02031-

1

Atokatheridium -------------------------------------------------------------

Sulestes -----------------------------------------------------0--2----

Asiatherium 11-------2--------------02011-2-00--21--100-0-0-2----0-020---

1

Kokopellia -------------------------------------------------------------

Anchistodelphys -------------------------------------------------------------

Albertatherium -------------------------------------------------------------

Didelphodon 1---------1-1-----------0------------------------------0---1-

Pediomys 1-----------1-----------------2---2------------------------1-

Turgidodon 1-----------1----------------------------------------------1-

Mayulestes 111------21-------------02011020002021101001000-201010-020--0

Pucadelphys 111------21-1------2----02011-2000202110100111012010100020310

Andinodelphys 11-------21-1-----------02-111-1002021-010011001201-10-020310

Didelphis 1112131112121011100221200201112100202100100110012010100020310

Marmosa 1112131112121011100221200201112100202100100110112010100020311

Caenolestes 1112131--21110111003212002011131002021-010011-01201010002032-

Dasyurus 121-131--2111011100421200201111120202110100110012010100020320

Perameles 1112131--2111011100421200201112100202100100110212010100020321

Dromiciops 1102131--21220111003213002011111002031101001100120-0100020321

Thylacomyidae 1--2131--21--0-1100421-0020111-1002021-010011-212-101-0020-2-

Macropus 1112131--2122011100521300201111110213110100110212010100030321

Acrobates 1202131--212-111100521310201113100203110100110012010100020321

Phascolarctos 1112131--2112011100321300201113110214100100111012010100030320

Vombatus 1112131--2112011100521300201113100214110100110012010100030320

Phalanger 1112131--2122111100521310201113110213100100110012010100030320

Pseudocheirus 1212131--2122111100521310201112100203110100110012010100120321

Petauroides 1212131--2122111100521310201112100202110100110012010100120321

Input data matrix (continued):

4444444444455555555555555555555555555555555555555

8999999999900000000001111111222222222233333333334

Taxon/Node 9012345678901234567890456789012345678901234567890

-------------------------------------------------------------------

Thrinaxodon 0000000000100000000000--03000-00000000000-000-000

Massetognathus 00000-000000000000001001120---00100000000-0000-00

Probainognathus 00000-0000000000000101--03000-000--000--0-000--00

Tritylodontids 00000000000000100-011100--001000100000--0-0100000

1 1

Pachygenelus 00000000000000000-------------0-0--00---0-010--00

Brasilitherium 0-0---0000-000-0--------0-000-0-0--0000000010--10

Brasilodon 000---0000-00--0--------0-0---0-0--000--0-010--10

Adelobasileus 1000-----------0----------0---0-0----0------0--1?

Sinoconodon 0000100000000010000101--03000-0-0--000--0-000--00

1

Morganucodon 1000110000000010000121--13000-000--000--00000-010

Megazostrodon 1-00-------00--0------------------------0-0----10

Haldanodon 1110110000000000---1----010---00000000--0-0-0--10

Castorocauda 1---11-00---------------------------------0---000

Docofossor -----1000-------------------------------0-----010

Agilodocodon -----1000-------------------------------0-0---010

Megaconus ----------------------00---0-------------------10

Cifelliodon 00100210110000-0001121-0120111111111110011111--0?

Millsodon -----------------------0-----------------------1?

Hahnodon -----------------------0-----------------------1?

Eleutherodon ----------------------00-----------------------1?

Sineleutherus ----------------------00-----------------------1?

Thomasia ----------------------11-----------------------1?

Haramiyavia -----1----------------11----------------0------10

Arboroharamiya ----------------------00-----------------------10

Xianshou linglon ----------------------00--011---0-------1----1-10

Xianshou songae ----------------------00------------------0--1110

Shenshou 2-0--1001------1------00---111--0---0---1-0--0110

Vilevolodon 10--0-00-------------------11-----------111--1110

Maiopatagium 1---0000--00----------------1------10-----0--011?

2

Vintana 00--1010-100-111110211---2011-11011111-011111--0?

2

Rugosodon ----------------------00----1-------------0--0010

Kuehneodon ---------1------------00------------------0--0-10

Sinobaatar ----------------------00------------------0--0-10

Plagiaulacids 0100011011001-21------00----------------0-0100-00

1 1

Cimolodontans 110012101000002111022100100110000-000011010100000

1 1 211 1 1 1

Hadrocodium 211001-00--000-2--1221----000-000---0010000-0--10

Shuotherium -----------------------------------------------10

Pseudotribos ----------------------------------------0------10

Asfaltomylos -----------------------------------------------10

Ambondro -----------------------------------------------10

Ausktribosphenos -----------------------------------------------10

Bishops -----------------------------------------------10

Teinolophos -----------------------------------------------10

Kryoryctes -----------------------------------------------0?

Steropodon -----------------------------------------------00

Obdurodon 2110011-1--00002----2---0-0--1000-000011010-0--01

Ornithorhynchus 2110011-1-100002111221--010--0000-000011010-0-101

Tachyglossus 2110011---100102111221--020---000-010011010-0-101

2

Fruitafossor ----------------------------------------0------10

Gobiconodon 110001-0-0001--0--0-00----0-----1-------0-0-0--00

Repenomamus 110001-0-0001010--0100--00----0-1-------0-0-0-000

Spinolestes ----------------------------------------0-0---010

Amphilestes ------------------------------------------0----10

Yanoconodon ----------------------------------------0-0---010

Jeholodens 11--01---------1----------0-------------0-0---010

Trioracodon ------------------------------------------0----10

Priacodon ---------0--0--1-------------------10----------10

Tinodon -----------------------------------------------10

Akidolestes ----0---0-------------------------------0-0---010

Spalacotherium -----------------------------------------------10

Zhangheotherium -------00-------------------------------0-0---010

Maotherium 1---0--00------1----------000---0----0--0-0-0-010

Dryolestes -------00--0------------------------------0----10

Henkelotherium ------------------------------------------0---010

Amphitherium -----------------------------------------------10

Peramus ------------------------------------------0----10

Vincelestes 0100111000000--1--------03000-0-2--000--0100--000

Nanolestes -----------------------------------------------10

Kielantherium -----------------------------------------------10

Aegialodon -----------------------------------------------1?

Montanalestes ---------------1-------------------------------10

Prokennalestes -----------0-----------------------------------10

Murtoilestes -----------------------------------------------1?

Eomaia ----02--1------1----------00000---------0-0-0-010

Juramaia --------------------------000-----------0-0----10

Kennalestes 1110021010-00--1011221-------0----------010-0--10

Asioryctes 1110021010000--1011221---30--0----------0-000-010

Ukhaatherium ---0------0---------------0-------------------010

Zalambdalestes 1100021010000--1011221--0000000-2--000--01000-010

Daulestes 1--00------00--2------------------------0-0----10

Aspanlestes -----------------------------------------------10

Eoungulatum -----------------------------------------------00

1

Cimolestes -----------------------------------------------00

1

Gypsonictops -----------------------------------------------10

Protungulatum ----------------------------------------------010

Erinaceus 1110021110020122011221--210001002-0000--01000-000

Leptictis 1110021010000122011221--2-0000002-0000--0-0-0-000

Canis 2100021111100122001221--2000000020000011010-0-000

1

Felis 2100021111100122001221--2000000020000011010-0-000

Rattus 210002121-200121111221--2001110000000011010-0-010

Oryctolagus 210002121-200121111221--2001110000000011110-0-000

Bradypus 21000210--001122001221--230---00200000110-100-000

Tamandua 21000210--001122001221--230---00200000--0---0-000

Glyptotherium 2101021011-01122---221--230--000200000--0-1-0-000

Dasypus 2101021011001122001221--230--000-0000011010-0-000

Chaetophractus 2101021011001122001221--230--000-00000--0-0-0-000

1

Euphractus 2101021011001122001221--230--000-00000--0-0-0-000

Holoclemensia -----------------------------------------------1?

Sinodelphys ----0---1------1-----------00-----------0-0-0-010

Deltatheridium ---002-011000-----------1-0--0-----00---0-0---010

Atokatheridium -----------------------------------------------1?

Sulestes -----------0-----------------------------------10

Asiatherium 11000------10--2----------0-------------------010

Kokopellia -----------------------------------------------10

Anchistodelphys -----------------------------------------------1?

Albertatherium -----------------------------------------------1?

Didelphodon ---0----1--1------------130000----------0-0-0-000

Pediomys -----------1----------------------------------010

Turgidodon -----------------------------------------------10

Mayulestes 1100021111100--2--------130000002-0000--------000

Pucadelphys 1100021011100-21011221--130000002000001101000-010

Andinodelphys 110002101111--21--------130000002-0000--0-0-0-010

Didelphis 0100021111110121011221--100000002000001101000-000

1

Marmosa 2110021111110121011221--13000000000000--0-000-010

Caenolestes -10002111-220121---221--13000000000000110-0-0-010

Dasyurus 1100021111110121011221--130000002000001101000-000

Perameles 2100021110110121---221--230001000-0000--0-000-010

Dromiciops 2110021011110121011221--13000000200000110-000-010

Thylacomyidae -1-0021-1---0121---221--230001000-0000--0-000-000

Macropus 211002101-000121---221--13000100200000111-000-000

1

2

Acrobates 2110021111120121---221--13000000000000110-000-010

Phascolarctos 1100021111020121---221--130111002000001101000-000

Vombatus 2100021111001121---221--130111002000001101000-000

Phalanger 2100021111120121---221--13011100200000110-000-000

Pseudocheirus 2100021111120121---221--13000100000000110-000-000

Petauroides 2110021011120121---221--10000000000000110-000-000

1. **Initial exploratory analyses**

72 of the multistate characters are ordered in some analyses:

charset ordered = 1 4 12 22 23 33 44 50 62 66 74 91 95 97 106 107 110 121 122 123 129 131-134 137 139 147 150 184 187 190-195 197 202 216 248 249 258 265 267 268 272 279 299 308 309 315 317 369 371 376 402 427 440 448 453 456 468 482 484 487 489 496 504 508 509 516;

***a) Initial maximum parsimony analyses***

Initial MP bootstrap analyses were also run on the 27 near-complete, dietarily plesiomorphic (NCDP) taxa and also on Australosphenida and separately for the multicuspate clades, Multituberculata and Haramiyida.

- NCDP_27_ (unordered)

(Thrinaxodon,Pachygenelus,(Sinoconodon,((Morganucodon,Megazostrodon)95,((Haldanodon,(Docofossor,Agilodocodon)100)100,((((Gobiconodon,Repenomamus)99,Spinolestes)86,((Zhangheotherium,Maotherium)100,(Henkelotherium,(Vincelestes,((((Ukhaatherium,Asioryctes)52,Kennalestes)95,(Zalambdalestes,Leptictis)69)100,(Deltatheridium,(Asiatherium,(Mayulestes,(Pucadelphys,Andinodelphys)97)86)99)97)100)77)88)100)80,(Yanoconodon,Jeholodens)49)100)99)100)100);

- NCDP_27_ (72 multistate characters ordered)

(Thrinaxodon,Pachygenelus,(Sinoconodon,((Morganucodon,Megazostrodon)95,((Haldanodon,(Docofossor,Agilodocodon)100)100,((((Gobiconodon,Repenomamus)99,Spinolestes)86,((Zhangheotherium,Maotherium)100,(Henkelotherium,(Vincelestes,((((Kennalestes,Asioryctes)51,Ukhaatherium)94,(Zalambdalestes,Leptictis)71)100,(Deltatheridium,(Asiatherium,(Mayulestes,(Pucadelphys,Andinodelphys)98)92)99)80)100)93)83)100)49,(Yanoconodon,Jeholodens)88)100)99)100)100);

- Australosphenida (unordered)

(Thrinaxodon,Pachygenelus,(Sinoconodon,((Morganucodon,Megazostrodon)99,(Asfaltomylos,(Ambondro,((Ausktribosphenos,Bishops)34,(Teinolophos,(Steropodon,(Kryoryctes,(Tachyglossus,(Obdurodon,Ornithorhynchus)56)49)39)44)67)63)64)96)82)98);

- Australosphenida (72 multistate characters ordered)

(Thrinaxodon,Pachygenelus,(Sinoconodon,((Morganucodon,Megazostrodon)99,(Asfaltomylos,(Ambondro,((Ausktribosphenos,Bishops)33,(Teinolophos,(Steropodon,(Kryoryctes,(Tachyglossus,(Obdurodon,Ornithorhynchus)54)47)38)47)73)70)68)95)94)98);

- Australosphenida without *Kryoryctes* (unordered)

(Thrinaxodon,Pachygenelus,(Sinoconodon,((Morganucodon,Megazostrodon)99,(Asfaltomylos,(Ambondro,((Ausktribosphenos,Bishops)36,(Teinolophos,(Steropodon,(Tachyglossus,(Obdurodon,Ornithorhynchus)70)80)54)85)71)69)100)86)99);

- Australosphenida without *Kryoryctes* (72 multistate characters ordered)

(Thrinaxodon,Pachygenelus,(Sinoconodon,((Morganucodon,Megazostrodon)99,(Asfaltomylos,(Ambondro,((Ausktribosphenos,Bishops)32,(Teinolophos,(Steropodon,(Tachyglossus,(Obdurodon,Ornithorhynchus)70)76)56)91)78)74)100)98)100);

-Multituberculata (unordered)

(Thrinaxodon,Pachygenelus,(Sinoconodon,((Morganucodon,Megazostrodon)85,(Cimolodontans,(Sinobaatar,(Plagiaulacids,(Kuehneodon,Rugosodon)100)99)82)100)99)100);

- Multituberculata (72 multistate characters ordered)

(Thrinaxodon,Pachygenelus,(Sinoconodon,((Morganucodon,Megazostrodon)90,(Cimolodontans,(Sinobaatar,(Plagiaulacids,(Kuehneodon,Rugosodon)95)88)60)100)100)100);

- Multituberculata no *Kuehneodon* (unordered)

(Thrinaxodon,Pachygenelus,(Sinoconodon,((Morganucodon,Megazostrodon)84,(Cimolodontans,(Sinobaatar,(Plagiaulacids,Rugosodon)82)72)100)99)100);

- Multituberculata no *Kuehneodon* (72 multistate characters ordered)

(Thrinaxodon,Pachygenelus,(Sinoconodon,((Morganucodon,Megazostrodon)87,(Cimolodontans,(Sinobaatar,(Plagiaulacids,Rugosodon)62)54)100)100)100);

- Haramiyida (unordered)

(Thrinaxodon,Pachygenelus,(Sinoconodon,((Morganucodon,Megazostrodon)95,(Haramiyavia,(Megaconus,((Cifelliodon,Vintana)84,((((Arboroharamiya,Vilevolodon)82,(Xianshou_linglong,Xianshou_songae)42)89,Shenshou)88,Maiopatagium)68)88)98)100)99)99);

- Haramiyida (72 multistate characters ordered)

(Thrinaxodon,Pachygenelus,(Sinoconodon,((Morganucodon,Megazostrodon)98,(Thomasia,(Haramiyavia,(Megaconus,((Hahnodontidae,Vintana)75,((((Arboroharamiya,Vilevolodon)86,(Xianshou_linglong,Xianshou_songae)42)89,Shenshou)87,Maiopatagium)89)82)92)69)99)100)100);

**Supplementary Table S1.** Comparisons of maximum parsimony bootstrap support values between the unordered and ordered analyses. Values are avergaes across groupings that are resolved in at least one of the ordered/unordered treatments with between 60-95% bootstrap probability (BP).

|  | **unordered** | **ordered** |
| --- | --- | --- |
| NCDP | 84.9 | 85.6 |
| Australosphenida | 72.5 | 76.5 |
| Australosphenida minus *Kryoryctes* | 75 | 77.8 |
| Multituberculata + Haramiyida | 79.6 | 79.5 |

***b ) Maximum Likelihood analyses results for NCDP_27_ and 53 taxon (53_537_) datasets***

- NCDP_27_ (unordered, 3 partitions, -sp):

(Thrinaxodon:0.0984757638,Pachygenelus:0.0666139450,(Sinoconodon:0.0397618857,((Morganucodon:0.0054938663,Megazostrodon:0.0308681051)95:0.0134208761,((Haldanodon:0.0098745637,(Docofossor:0.0269763357,Agilodocodon:0.0397396229)100:0.0543523373)100:0.1122092073,(((((Gobiconodon:0.0054587171,Repenomamus:0.0033877268)99:0.0527819065,Spinolestes:0.0633115670)99:0.0877059890,((Zhangheotherium:0.0000022575,Maotherium:0.0140268861)99:0.0716022924,(Henkelotherium:0.0164871692,(Vincelestes:0.0852806130,((((Kennalestes:0.0306871030,Asioryctes:0.0036486197)76:0.0170392655,Ukhaatherium:0.0070110757)83:0.0264199227,(Zalambdalestes:0.0444649068,Leptictis:0.1338985320)96:0.0419133917)100:0.1182345261,(Deltatheridium:0.0314525010,(Asiatherium:0.0379476419,(Mayulestes:0.0249229125,(Pucadelphys:0.0032441802,Andinodelphys:0.0218555099)98:0.0238359682)79:0.0455244230)99:0.0753769542)97:0.0957691409)100:0.2161798495)90:0.0681316778)99:0.1084745809)100:0.2362418398)66:0.0585557398,Yanoconodon:0.0407252563)49:0.0149325411,Jeholodens:0.0000023669)100:0.2422296531)100:0.0617442339)100:0.1129839104)100:0.0708115353);

- NCDP_27_ (ordered, 6 partitions, -sp):

(Thrinaxodon:0.1107967577,Pachygenelus:0.0752182896,(Sinoconodon:0.0717021841,((Morganucodon:0.0091247302,Megazostrodon:0.0261699982)99:0.0425408952,((Haldanodon:0.0084807174,(Docofossor:0.0586508122,Agilodocodon:0.0696966779)100:0.0757206183)100:0.0926164118,(((((Gobiconodon:0.0054444445,Repenomamus:0.0033261133)100:0.0717388104,Spinolestes:0.0576928321)100:0.0830048887,((Zhangheotherium:0.0000024573,Maotherium:0.0183422944)100:0.0919988231,(Henkelotherium:0.0131028020,(Vincelestes:0.1209968816,((((Kennalestes:0.0292420839,Asioryctes:0.0047768958)63:0.0169357551,Ukhaatherium:0.0128502933)92:0.0256426886,(Zalambdalestes:0.0475002874,Leptictis:0.1310379146)94:0.0382552263)100:0.1551696604,(Deltatheridium:0.0376640840,(Asiatherium:0.0331293773,(Mayulestes:0.0264988432,(Pucadelphys:0.0032656426,Andinodelphys:0.0216159186)95:0.0210797105)62:0.0529017158)97:0.0719444895)98:0.0717329020)100:0.1903212974)94:0.1271830476)100:0.1088784884)100:0.2887460732)55:0.0637931194,Yanoconodon:0.0346827634)48:0.0167614727,Jeholodens:0.0019697369)100:0.3151064477)100:0.0578170260)100:0.1366017244)100:0.0726707049);

- NCDP_27_ (ordered, 6 partitions -spp):

(Thrinaxodon:0.1532529476,Pachygenelus:0.0479144261,(Sinoconodon:0.0426732457,((Morganucodon:0.0105051079,Megazostrodon:0.0258034604)90:0.0310979939,((Haldanodon:0.0106554371,(Docofossor:0.0553812704,Agilodocodon:0.0531156674)100:0.0673437707)100:0.1060425117,(((((Gobiconodon:0.0052046501,Repenomamus:0.0031128996)96:0.0520103378,Spinolestes:0.0825544160)97:0.1422763425,Yanoconodon:0.0302315176)37:0.0278314103,Jeholodens:0.0060060463)51:0.0783656699,((Zhangheotherium:0.0000026500,Maotherium:0.0121094912)100:0.1003404190,(Henkelotherium:0.0095142184,(Vincelestes:0.1030870810,((((Kennalestes:0.0348262154,Asioryctes:0.0000029956)73:0.0184186738,Ukhaatherium:0.0061603553)81:0.0223216417,(Zalambdalestes:0.0421661692,Leptictis:0.1445023468)95:0.0464151042)100:0.1447884931,(Deltatheridium:0.0263121235,(Asiatherium:0.0377260531,(Mayulestes:0.0179183972,(Pucadelphys:0.0027705204,Andinodelphys:0.0226695266)98:0.0307560844)65:0.0384651311)99:0.0988407746)98:0.1000960456)100:0.2286746063)95:0.0943982820)98:0.1069137249)100:0.2989521525)100:0.2238340748)100:0.0808418512)100:0.1345055394)100:0.1095422116);

- *53_537_* (unordered, 3 partitions, -sp):

(Thrinaxodon:0.0975843107,Pachygenelus:0.0681200982,(Sinoconodon:0.0357794209,((Morganucodon:0.0052309609,Megazostrodon:0.0298835014)99:0.0323612749,(((Haldanodon:0.0170989646,(Docofossor:0.0257384318,Agilodocodon:0.0394551958)100:0.0425068230)100:0.1102920436,((((((((Rugosodon:0.0114604771,Kuehneodon:0.0123267531)97:0.0381300438,Plagiaulacids:0.0742087720)97:0.0391019887,Sinobaatar:0.0000023697)97:0.0340005014,Cimolodontans:0.0217421922)98:0.3257933400,((Zhangheotherium:0.0000026182,Maotherium:0.0146787377)100:0.0775218078,(Henkelotherium:0.0133778241,(Vincelestes:0.0893075736,((((Kennalestes:0.0302275992,Asioryctes:0.0066525097)91:0.0151895949,Ukhaatherium:0.0095987416)93:0.0254188110,(Zalambdalestes:0.0439329120,Leptictis:0.1333989492)99:0.0404216178)100:0.1119255605,(Deltatheridium:0.0321146888,(Asiatherium:0.0383200332,(Mayulestes:0.0248305034,(Pucadelphys:0.0026256657,Andinodelphys:0.0219270413)100:0.0242404154)94:0.0452625904)100:0.0741737193)100:0.0995455266)100:0.2014481493)95:0.0703611021)98:0.0973589229)97:0.1333687077)74:0.0913227934,((Gobiconodon:0.0054882729,Repenomamus:0.0033604660)99:0.0504686013,Spinolestes:0.0647108230)86:0.1094595613)33:0.0477855331,(Yanoconodon:0.0410643508,Jeholodens:0.0140002813)37:0.0163023465)42:0.1155411169,(Asfaltomylos:4.5409433747,(Ambondro:0.0069717502,((Ausktribosphenos:0.0791422904,((Teinolophos:0.5577379007,((Obdurodon:0.0043219473,Ornithorhynchus:0.0129772461)33:0.0160998508,Tachyglossus:0.0752380842)74:0.0195005182)39:0.0056119098,(Steropodon:0.0000017342,Kryoryctes:0.0251899519)11:0.0148705238)82:0.2064532205)40:0.0148842739,Bishops:0.0056334681)73:0.0837690547)72:0.0539522137)78:0.3619973497)84:0.0980878575)58:0.0333462140,((Megaconus:0.0824874517,((Hahnodontidae:0.0649332986,Vintana:0.2166866492)91:0.1555486007,((((Arboroharamiya:0.0252740453,Vilevolodon:0.0298547825)93:0.0301087978,(Xianshou_linglong:0.0000024022,Xianshou_songae:0.0485275193)99:0.0090728635)97:0.0502946931,Shenshou:0.0108367536)96:0.0463290288,Maiopatagium:0.0367658934)85:0.0628682309)84:0.1650231431)80:0.0998800361,(Thomasia:0.0293826386,Haramiyavia:0.0107706311)95:0.0604977547)77:0.1138075792)99:0.0465372728)100:0.0922430848)100:0.0713091821);

- *53_537_* (ordered, 6 partitions, -sp):

(Thrinaxodon:0.1123817208,Pachygenelus:0.0846707747,(Sinoconodon:0.0719288069,((Morganucodon:0.0098941916,Megazostrodon:0.0264915046)100:0.0386797450,(((Haldanodon:0.0207850085,(Docofossor:0.0609140964,Agilodocodon:0.0716740134)100:0.0617170304)100:0.0845145782,(((((Vintana:0.2691123042,((((Rugosodon:0.0112256183,Kuehneodon:0.0133802995)100:0.0438409265,Plagiaulacids:0.0688533548)100:0.0349612723,Sinobaatar:0.0000022867)100:0.0327966642,Cimolodontans:0.0244705831)98:0.0925240668)74:0.2536656988,((Zhangheotherium:0.0000022157,Maotherium:0.0244929279)100:0.1016074235,(Henkelotherium:0.0141277199,(Vincelestes:0.1225908063,((((Kennalestes:0.0301644469,Asioryctes:0.0075507633)81:0.0134666859,Ukhaatherium:0.0151200558)100:0.0263727738,(Zalambdalestes:0.0487578399,Leptictis:0.1314185478)99:0.0381303032)100:0.1491720437,(Deltatheridium:0.0401273669,(Asiatherium:0.0340961854,(Mayulestes:0.0270134471,(Pucadelphys:0.0040935561,Andinodelphys:0.0218285119)99:0.0203461349)72:0.0526870760)99:0.0715547622)99:0.0717069792)100:0.1908353682)86:0.1261129590)91:0.0947778626)90:0.1888381248)81:0.0965379158,((Gobiconodon:0.0056118873,Repenomamus:0.0033906982)100:0.0703121415,Spinolestes:0.0590236067)100:0.1117857574)39:0.0404043971,(Yanoconodon:0.0404994809,Jeholodens:0.0132040800)59:0.0303249946)55:0.0876435114,(Asfaltomylos:0.6114300966,(Ambondro:0.0064988050,((Ausktribosphenos:0.0344761338,Bishops:0.0050289887)49:0.0000024551,((Teinolophos:0.7147132044,(((Obdurodon:0.0053102551,Kryoryctes:0.0208820747)15:0.0000044015,Ornithorhynchus:0.0106241812)14:0.0169684533,Tachyglossus:0.0834231740)29:0.0521512636)58:0.0183301761,Steropodon:0.0000020983)98:0.2347607813)91:0.0862509155)91:0.0496950407)91:0.4124758141)84:0.1998315059)66:0.0444770129,((Megaconus:0.0841861321,(Hahnodontidae:0.2263332151,((((Arboroharamiya:0.0252691050,Vilevolodon:0.0429201770)97:0.0181385779,(Xianshou_linglong:0.0000024851,Xianshou_songae:0.0209651963)100:0.0095383102)98:0.0483656474,Shenshou:0.0107620707)97:0.0484521906,Maiopatagium:0.0515701151)57:0.0436752466)84:0.1869751534)81:0.1377382824,(Thomasia:0.0211804780,Haramiyavia:0.0443963801)99:0.0487421551)83:0.2514279538)99:0.0356385567)100:0.1375300386)100:0.0740047201);

- *53_537_* (ordered, 6 partitions -spp):

(Thrinaxodon:0.1534086478,Pachygenelus:0.0500629509,(Sinoconodon:0.0437552510,((Morganucodon:0.0112394244,Megazostrodon:0.0249571385)96:0.0320569368,((Haldanodon:0.0189249738,(Docofossor:0.0537793978,Agilodocodon:0.0548481284)99:0.0576690655)99:0.1267084500,(((Megaconus:0.1469255972,((Hahnodontidae:0.0921701498,Vintana:0.3121576051)97:0.1810943285,((((Arboroharamiya:0.0241266785,Vilevolodon:0.0249272418)94:0.0246079340,(Xianshou_linglong:0.0000026733,Xianshou_songae:0.0000020085)99:0.0145401878)99:0.0485547702,Shenshou:0.0095719403)96:0.0324554455,Maiopatagium:0.0345778675)94:0.1036127869)93:0.1233620566)88:0.1037304577,(Thomasia:0.0620207647,Haramiyavia:0.0038077299)95:0.0936815524)89:0.1092571874,(((((((((Rugosodon:0.0150790098,Kuehneodon:0.0166053709)99:0.0506130394,Plagiaulacids:0.0052845291)99:0.0283158158,Sinobaatar:0.0000020802)98:0.0204487085,Cimolodontans:0.0416123302)100:0.2011083450,((((Asfaltomylos:0.0961590262,Ambondro:0.0089037937)77:0.1632848199,Bishops:0.0000020400)73:0.0144775276,Ausktribosphenos:0.0000023703)78:0.1469715735,(Teinolophos:0.0112170697,(Steropodon:0.0101409840,(Obdurodon:0.0000023923,(Ornithorhynchus:0.0028392675,Tachyglossus:0.0593672340)73:0.0194513630)95:0.0279538563)83:0.0552186653)88:0.0936351646)87:0.3468423506)38:0.1299593660,((Zhangheotherium:0.0000026921,Maotherium:0.0124830656)100:0.0841839203,(Henkelotherium:0.0357831326,(Vincelestes:0.1013651214,((((Kennalestes:0.0355446517,Asioryctes:0.0000028842)95:0.0188808834,Ukhaatherium:0.0062220248)99:0.0228535759,(Zalambdalestes:0.0433673480,Leptictis:0.1433449728)96:0.0456001745)100:0.1373306001,(Deltatheridium:0.0297683366,(Asiatherium:0.0376202429,(Mayulestes:0.0187216320,(Pucadelphys:0.0027958963,Andinodelphys:0.0231192690)99:0.0305933105)84:0.0386900986)100:0.0951292643)100:0.1039774407)100:0.2250368689)93:0.0814992214)96:0.1102126386)96:0.1294289559)70:0.1775929296,((Gobiconodon:0.0057385111,(Repenomamus:0.0028683992,Kryoryctes:0.1435947285)45:0.0000021005)88:0.0572253737,Spinolestes:0.0798056559)88:0.1221018165)37:0.0474726447,Yanoconodon:0.0388783825)41:0.0251756893,Jeholodens:0.0000021890)92:0.2164083541)84:0.0711776785)99:0.0651291691)100:0.1318386571)100:0.1095025173);

**Supplementary Table S2.** Comparison of alternative partitioning strategies in IQ-TREE for the 53_537_ and 53_350_ datasaets, showing tree likelihoods, corrected Akaike information criterion scores and Bayesian information criterion scores. Characters were either all unordered or ordered for only the 72 multistate characters that were identified as appropriate to order. -sp allows branch-length estimates to be fully independent between partitions, while for -spp the overall rates can vary between partitions, but the relative branch-lengths are fixed.

|  | **logL** | | **AICc** | **BIC** |
| --- | --- | --- | --- | --- |
| 53_537_ dataset (all characters) | |  | | |
| unordered -sp | -8430.2836 | | 18126.6648 | 18683.5067 |
| ordered/unordered -sp | -8078.5163 | | 654605.0326 | 19702.3355 |
| ordered/unordered -spp | -8429.9243 | | 17149.9813 | 17576.4524 |
| 53_350_ dataset (minus cheek and shoulder girdle characters) | | | | |
| Unordered -sp | -5179.89 | | 13537.55 | 12035.14 |
| ordered/unordered -sp | -4950.04 | | 625948.1 | 13145.37 |
| ordered/unordered -spp | -5225.08 | | 10780.96 | 11106.24 |

1. **Testing phylogenetic congruence between anatomical regions**
2. ***Maximum likelihood trees for the NCDP_27_ dataset***

- Mandibulodental partition (unordered -sp):

(Thrinaxodon:0.0669629223,Pachygenelus:0.1480781721,(Sinoconodon:0.0622136704,((Morganucodon:0.0000022479,(Megazostrodon:0.0329697200,(((((Gobiconodon:0.0078303319,Repenomamus:0.0082685891)100:0.1057217274,Spinolestes:0.0665365515)94:0.0240514206,((Zhangheotherium:0.0000021125,Maotherium:0.0067165549)100:0.0862124522,(Henkelotherium:0.0370418645,(Vincelestes:0.0829485041,((((Kennalestes:0.0327677887,(Zalambdalestes:0.0789196812,Leptictis:0.1391983851)100:0.1013219066)47:0.0397655654,Asioryctes:0.0000021927)39:0.0231872947,Ukhaatherium:0.0080272147)100:0.2167964290,(Deltatheridium:0.0000024066,((Asiatherium:0.0658649873,(Pucadelphys:0.0000021125,Andinodelphys:0.0224287770)100:0.0307405172)63:0.0463992099,Mayulestes:0.0035774483)100:0.1787132327)100:0.1202358556)100:0.2853672975)80:0.0926426359)98:0.1814308301)100:0.2811287081)90:0.1311586654,Jeholodens:0.0000024665)70:0.0458674025,Yanoconodon:0.0664534080)99:0.2942084209)36:0.0429429324)29:0.0220626745,(Haldanodon:0.0124905599,(Docofossor:0.0518108879,Agilodocodon:0.0709476721)100:0.0839629156)100:0.2217983209)100:0.1614956797)100:0.0604246073);

- Cranial partition (unordered -sp):

(Thrinaxodon:0.1713837160,Pachygenelus:0.0077704532,(Sinoconodon:0.0253966076,((Morganucodon:0.0160188588,Megazostrodon:0.0000023383)76:0.0137249297,((Haldanodon:0.0000023675,(Docofossor:0.0000023383,Agilodocodon:0.0000023383)100:0.0000023383)100:0.0441763391,((((Gobiconodon:0.0000023383,Repenomamus:0.0000023383)99:0.0263979108,Spinolestes:0.0239683774)95:0.1342430709,(Yanoconodon:0.0000023518,Jeholodens:0.0000020105)70:0.0288191825)58:0.1032911737,((Zhangheotherium:0.0000023383,Maotherium:0.0318857170)100:0.0692885863,(Henkelotherium:0.0184592148,(Vincelestes:0.0847447728,((((Kennalestes:0.0000023383,Asioryctes:0.0000023383)89:0.0178292285,Ukhaatherium:0.0024356693)71:0.0221408701,(Zalambdalestes:0.0147739464,Leptictis:0.1563406188)100:0.0000026955)100:0.1109436323,(Deltatheridium:0.0212718515,(Asiatherium:0.0666165716,(Mayulestes:0.0191532617,(Pucadelphys:0.0068455681,Andinodelphys:0.0370443493)100:0.0128389820)70:0.0345852468)100:0.0284299576)100:0.0973897369)100:0.1943992709)74:0.0486723794)48:0.0507632422)59:0.0875044552)99:0.1454332515)97:0.0738070115)100:0.0908464843)100:0.1351173020);

- Postcranial partition (unordered -sp):

((((((((((((Asiatherium:0.0382116567,(Mayulestes:0.0074977119,(Pucadelphys:0.0701520266,Andinodelphys:0.0074977119)100:0.0701520266)91:0.1470521672)100:0.2027591206,Deltatheridium:0.0074977119)100:0.2027591206,(((Kennalestes:0.0074977119,Asioryctes:0.0150663587)70:0.0150663587,Ukhaatherium:0.0074977119)85:0.0304219165,(Zalambdalestes:0.0150663587,Leptictis:0.1034208536)100:0.0540224480)100:0.0949730455)100:0.1381334573,Vincelestes:0.0540224480)73:0.1119588216,Henkelotherium:0.0382116567)70:0.1205888945,(Zhangheotherium:0.0150663587,Maotherium:0.0074977119)100:0.0949730455)100:0.3052006621,(((Gobiconodon:0.0074977119,Repenomamus:0.0074977119)100:0.0150663587,Spinolestes:0.0866135128)100:0.0783404297,Yanoconodon:0.0074977119)97:0.0460779934)71:0.0382116567,Jeholodens:0.0304219165)100:0.1744208568,(Haldanodon:0.0074977119,(Docofossor:0.0227072955,Agilodocodon:0.0382116567)100:0.1034208536)100:0.0949730455)100:0.1293130812,(Morganucodon:0.0074977119,Megazostrodon:0.0074977119)96:0.0304219165)84:0.0540224480,Sinoconodon:0.0074977119)100:0.0227072955,Thrinaxodon:0.0460779934,Pachygenelus:0.0227072955);

1. ***51_537_ dataset***

“basic” constraint used in Figure 2b:

(Thrinaxodon,Pachygenelus,(Sinoconodon,(Morganucodon,Megazostrodon,((Docofossor,Agilodocodon),Haldanodon),(Repenomamus,Gobiconodon),Spinolestes,Jeholodens,Yanoconodon,(Zhangheotherium,Maotherium),Henkelotherium,Vincelestes,(Kennalestes,Asioryctes,Ukhaatherium,Zalambdalestes,Leptictis),Deltatheridium,(Asiatherium,Mayulestes,(Pucadelphys,Andinodelphys)),(Cimolodontans,Sinobaatar,Plagiaulacids,Rugosodon,Kuehneodon),(Asfaltomylos,Ambondro,Ausktribosphenos,Bishops,Teinolophos,Steropodon,Kryoryctes,Obdurodon,Ornithorhynchus,Tachyglossus),Haramiyavia,Thomasia,Megaconus,Arboroharamiya,Vilevolodon,Xianshou_linglong,Xianshou_songae,Shenshou,Maiopatagium)));

Constraint used in congruence analyses:

(Thrinaxodon,Pachygenelus,(Sinoconodon,(Morganucodon,Megazostrodon),((Docofossor,Agilodocodon),Haldanodon),((Repenomamus,Gobiconodon),Spinolestes),Jeholodens,Yanoconodon,(Zhangheotherium,Maotherium),Henkelotherium,Vincelestes,((Kennalestes,Asioryctes,Ukhaatherium),(Zalambdalestes,Leptictis)),(Deltatheridium,(Asiatherium,(Mayulestes,(Pucadelphys,Andinodelphys)))),(Cimolodontans,Sinobaatar,(Plagiaulacids,(Rugosodon,Kuehneodon))),(Asfaltomylos,Ambondro,(Ausktribosphenos,Bishops,(Teinolophos,Steropodon,Kryoryctes,(Obdurodon,Ornithorhynchus,Tachyglossus)))),(Haramiyavia,Thomasia,(Megaconus,((((Arboroharamiya,Vilevolodon),(Xianshou_linglong,Xianshou_songae)),Shenshou),Maiopatagium)))));


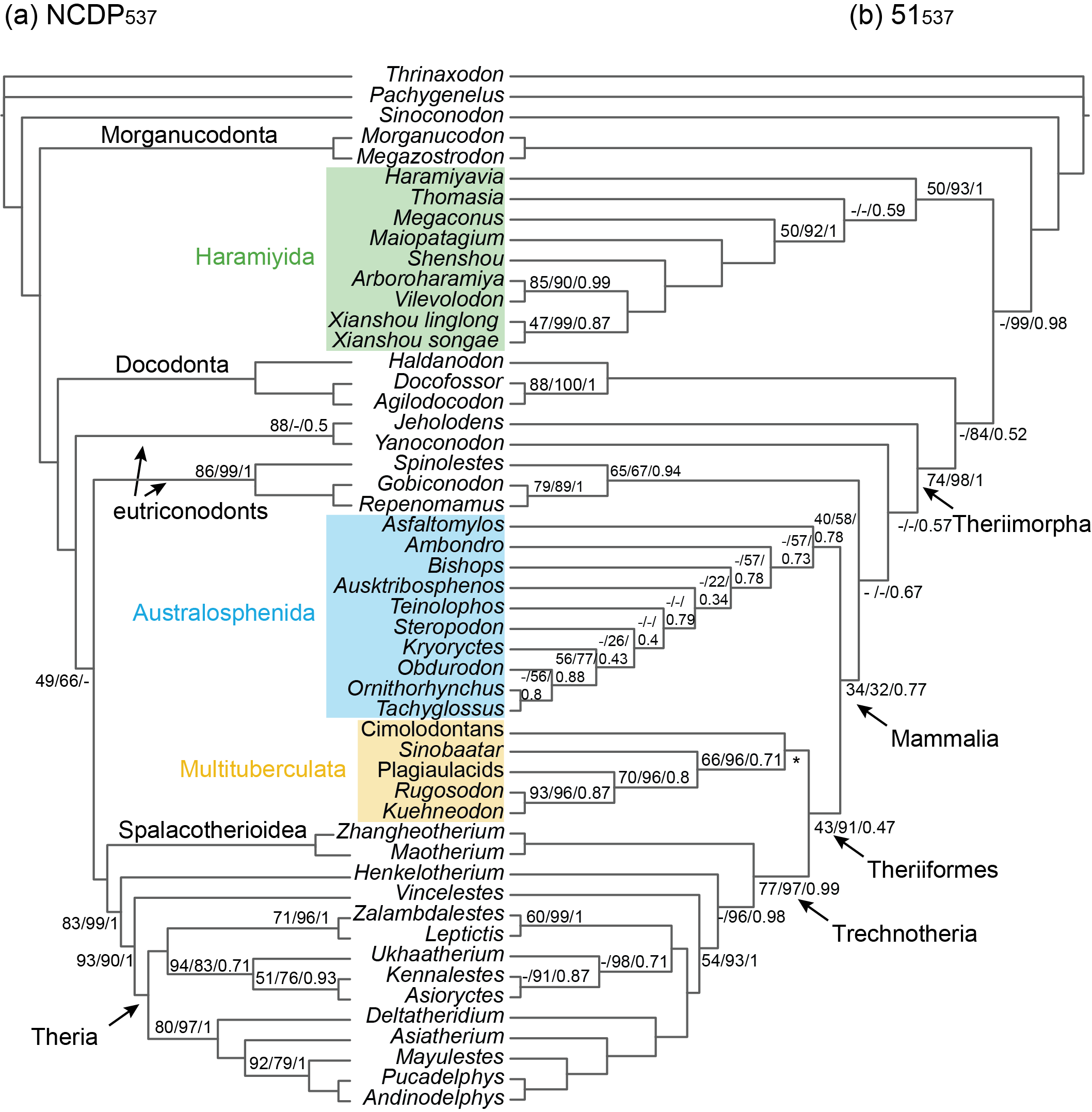


**Supplementary Figure S1**. (a) NCDP_537_, (b) 51_537_ Bayesian inference phylogenies reconstructed without topological constraint. Support values are represented at nodes, respectively for MP-ordered, ML and BI when <95%. Dashes represent branches not supported.

1. **Homoplasy within anatomical regions**

**Supplementary Table S3.** Maximum parsimony (MP) disadvantage for each sub-region indicating relative levels of correlated homoplasy attributable to including the ecologically apomorphic australosphenidans, multituberculates and haramiyids with the generalized insectivores. Homoplasy index is also given for each subregion on the overall 53_537_ MP trees. Corrected MP disadvantage ratios differ slightly from those in Fig. 6, because the expected (power) curve regression is based on three additional data points here, the axial, forelimb and pelvic girdle sub-regions that were excluded in the primary analysis, due to those sub-regions sampling few (<20) characters.

|  | Characters number | MP disadvantage (%) | Corrected MP disadvantage (ratios) relative to expected curve | HI (rank) |
| --- | --- | --- | --- | --- |
| Cheek teeth | 163 | 7.3 | 1.850166 | 0.547 (5) |
| Other dental | 23 | 8.8 | 0.782302 | 0.637 (1) |
| Mandibular | 34 | 12 | 1.314891 | 0.575 (4) |
| Basicranial | 117 | 4.4 | 0.933898 | 0.408 (10) |
| Calvariaviscerocranial | 68 | 3.2 | 0.508054 | 0.577 (3) |
| Axial | 16 | 22.2 | 1.625267 | 0.514 (6) |
| Shoulder girdle | 24 | 20 | 1.818907 | 0.5 (7) |
| Forelimb | 17 | 12.7 | 0.96042 | 0.621 (2) |
| Pelvic girdle | 13 | 8 | 0.524104 | 0.44 (9) |
| Hindlimb | 62 | 4 | 0.604445 | 0.453 (8) |


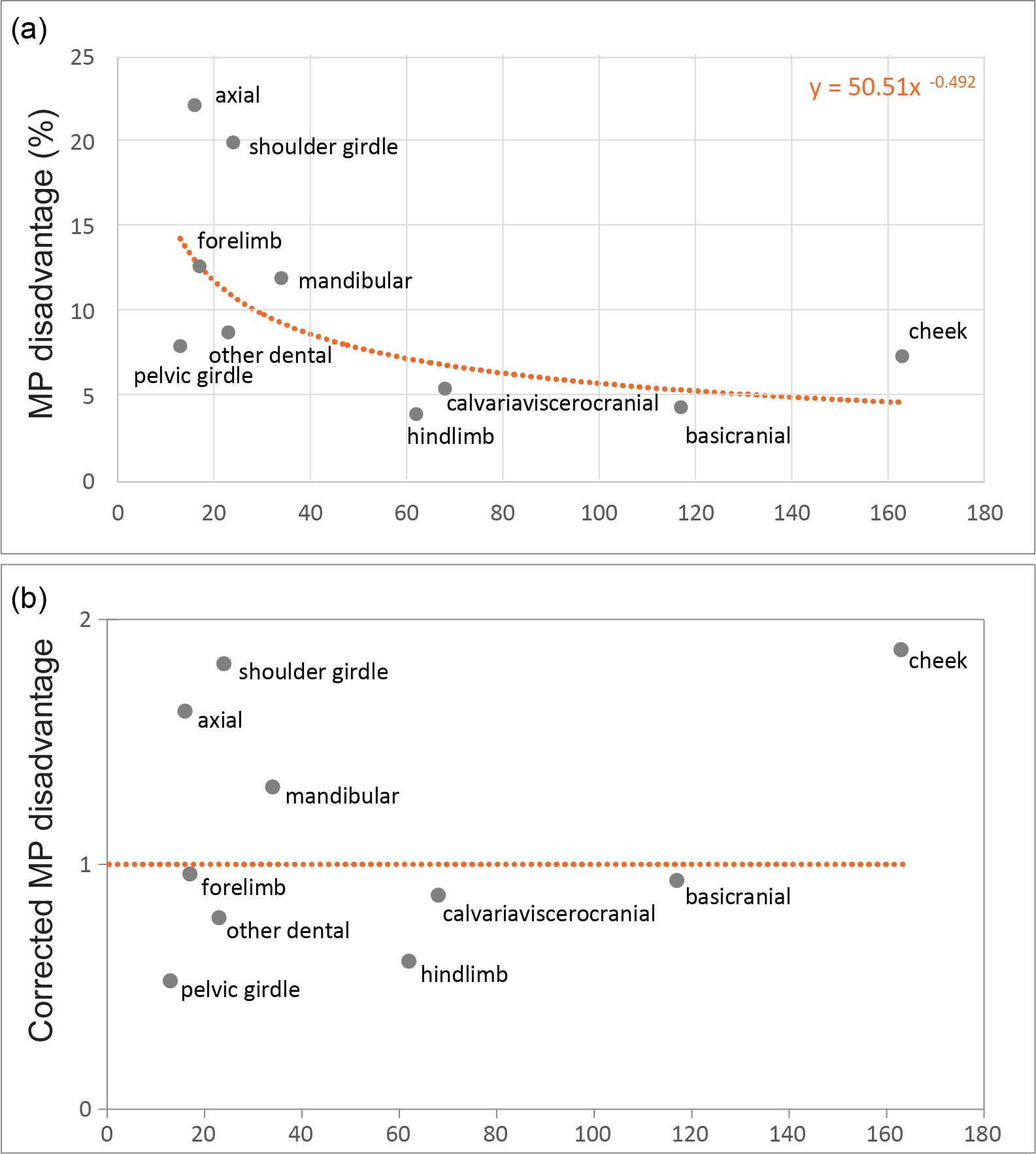


**Supplementary Figure S2**. Maximum parsimony disadvantage for each sub-region (a) expressed as a percentage and regressed across sub-regions as a power curve and (b) corrected maximum parsimony disadvantage for those same values, but compared as a ratio relative to their expected values from the power curve regression. Cheek teeth and shoulder girdle sub-regions are contributing excess correlated homoplasy relative to the expectation curve. Power curve, Y=50.51x^-0.492^

1. **Incongruence analyses and correlated homoplasy reduction**

**Supplementary Table S4.** Kishino-Hasegawa tests in IQ-TREE assessing congruence between the overall 51_537_, and 51_350_ topologies on the mandibulodental, cranial and postcranial data. Favoured (fav) uses the unconstrained topology, adjusted (adj) constrains for taxa along backbone phylogeny (Figure S1a)

|  | **51_537_** | | **51_350_** | |
| --- | --- | --- | --- | --- |
| Matrix used | *P*-value | Δln*L* | *P*-value | Δln*L* |
| Mandibulodental fav | **0.0003** | 84.468 | 0.0827 | 14.401 |
| Mandibulodental adj | **0.0005** | 82.768 | 0.0913 | 10.638 |
|  | | | | |
| Cranial fav | **0.0289** | 21.766 | **0.0454** | 17.818 |
| Cranial adj | **0.0301** | 21.766 | 0.0696 | 15.311 |
|  | | | | |
| Postcranial fav | 0.243 | 7.2297 | 0.699 | **0** |
| Postcranial adj | 0.498 | 0.186 | 0.202 | 4.2128 |

1. **References**

Bi, S., Wang, Y., Guan, J., Sheng, X., Meng, J. (2014). Three new Jurassic euharamiyidan species reinforce early divergence of mammals. *Nature*, *514*, 579–584.

Huttenlocker, A. K., Grossnickle, D. M., Kirkland, J. I., Schultz, J. A., Luo, Z. X. (2018). Late-surviving stem mammal links the lowermost cretaceous of North America and Gondwana. *Nature, 558*, 108–123.

Luo, Z.-X. (2015). Origin of the mammalian shoulder. In K. P. Dial, N. H. Shubin, & E. L. Brainerd (Eds.), *Great Transformations: Major Events in the History of Vertebrate Life* (pp. 167–187). University of Chicago Press.

Luo, Z.-X., Gatesy, S. M., Jenkins, F. A., Amaral, W. W., Shubin, N. H. (2015). Mandibular and dental characteristics of Late Triassic mammaliaform *Haramiyavia* and their ramifications for basal mammal evolution. *Proceedings of the National Academy of Sciences*, *112*, E7101–E7109.

Phillips, M. J., Bennett, T. H., Lee, M. S. Y. (2009). Molecules, morphology, and ecology indicate a recent, amphibious ancestry for echidnas. *Proceedings of the National Academy of Sciences of the United States of America*, *106*, 17089–17094.

Pridmore, P. A., Rich, T. H., Vickers-Rich, P., Gambaryan, P. P. (2005). A tachyglossid-like humerus from the early cretaceous of South-Eastern Australia. *Journal of Mammalian Evolution*, *12*, 359–378.

Ramírez-Chaves, H. E., Weisbecker, V., Wroe, S., Phillips, M. J. (2016). Resolving the evolution of the mammalian middle ear using Bayesian inference. *Frontiers in Zoology, 13*, 1–10.

Rich, T. H., Hopson, J. A., Gill, P. G., Trusler, P., Rogers-Davidson, S.. Morton, S., Cifelli, R. L., Pickering, D., Kool, L., Siu, K., Burgmann, F. A., Senden, T., Evans, A. R., Wagstaff, B. E., Seegets-Villiers, D, Corfe, I. J., Flannery, T. F., Walker, K., Musser, A. M., Archer, M., Pian, R. and P. Vickers-Rich. (2016). The mandible and dentition of the Early Cretaceous monotreme *Teinolophos trusleri*, *Alcheringa:* *An Australasian Journal of Palaeontology, 40*: 475-501

Rougier, G. W., Apesteguía, S., Gaetano, L. C. (2011). Highly specialized mammalian skulls from the Late Cretaceous of South America. *Nature, 479*, 98.

Zhou, C.-F., Wu, S., Martin, T., Luo, Z.-X. (2013). A Jurassic mammaliaform and the earliest mammalian evolutionary adaptations. *Nature*, *500*, 163–167.
